# MAOMAO: an ontology-guided FAIR resource for harmonized peptide toxicity data

**DOI:** 10.3389/fphar.2026.1952924

**Published:** 2026-10-16

**Authors:** Nicole Soto-Garcia, Julián García-Vinuesa, Roberto Uribe-Paredes, Leandro Murgas, Karen Oróstica, Jorge González-Puelma, Marcelo A. Navarrete, Frederic Cadet, David Medina-Ortiz

**Affiliations:** 1 Departamento de Ingeniería en Computación, Universidad de Magallanes, Punta Arenas, Chile; 2 Data Science Institute, Universidad del Desarrollo, Santiago, Chile; 3 Centro Asistencial Docente e Investigación, Universidad de Magallanes, Punta Arenas, Chile; 4 Escuela de Medicina, Universidad de Magallanes, Punta Arenas, Chile; 5 PEACCEL, AI for Biologics, Paris, France

**Keywords:** data integration, FAIR resource, machine learning, ontology-guided harmonization, peptide toxicity, predictive toxicology

## 1 Introduction

Therapeutic peptides have emerged as a rapidly expanding class of biomolecules with applications spanning antimicrobial, antiviral, anticancer, immunomodulatory, metabolic, and diagnostic settings (Goles et al., 2024). Their high target specificity, favorable safety profiles, and growing clinical success have positioned them as attractive alternatives or complements to traditional small-molecule therapeutics (Wang et al., 2022). The number of experimentally characterized bioactive peptides has therefore increased substantially during the last decade (Xiao et al., 2025), supporting the development of specialized databases and computational resources for peptide discovery, functional annotation, and rational design (Cabas-Mora et al., 2024; Ma et al., 2025; Singh et al., 2026; Wang et al., 2016). Despite these advances, toxicity remains a major limitation to peptide development and clinical translation, constraining candidate selection during early-stage screening and optimization (Bahar et al., 2025).

Peptide toxicity encompasses a broad spectrum of adverse biological effects that arise from the interaction between peptides and biological systems. Most toxicity phenotypes are routinely evaluated during peptide development including cytotoxicity, hemolysis, neurotoxicity, embryotoxicity, and ichthyotoxicity (Moreno-Vargas and Prada-Gracia, 2024; Rathore et al., 2025a). These toxicity endpoints have direct implications for efficacy, safety, dosage, and regulatory approval. Toxicity annotations have consequently accumulated across public databases (Singh et al., 2026; Minkiewicz et al., 2019; Pirtskhalava et al., 2021), literature-derived resources (Rathore et al., 2024a; Rathore et al., 2024b; Rathore et al., 2025b; Wang et al., 2026), supplementary datasets (Karasev et al., 2024; Bh et al., 2024; Perveen et al., 2023), and experimental studies (Timmons and Hewage, 2020; Yang and Xu, 2024). However, these resources remain fragmented, employ heterogeneous terminology and annotation criteria, and provide variable levels of metadata and evidence quality (Ojeda et al., 2025). Their development for distinct biological objectives further complicates integration, reproducibility, and large-scale analysis (Medina-Ortiz et al., 2026d).

The growing availability of toxicity data creates opportunities for computational toxicology, data-driven peptide engineering, and large-scale biological analyses (Soto-Garcia et al., 2026). Their utility, however, depends on the quality, consistency, and transparency of the underlying data (Lones, 2024; Gebru et al., 2022). Annotation ambiguity, conflicting evidence, heterogeneous endpoint definitions, incomplete metadata, and limited provenance tracking can reduce reproducibility and complicate downstream reuse (Al Shareef et al., 2025). The FAIR principles—Findability, Accessibility, Interoperability, and Reusability—provide a framework for organizing and distributing scientific data to support discovery, integration, reproducibility, and long-term reuse (Perez-Riverol et al., 2025). Nevertheless, a standardized framework that harmonizes heterogeneous peptide-toxicity annotations while preserving evidence, provenance, and metadata remains largely unavailable (Ojeda et al., 2025). FAIR-oriented resources tailored to both biological interpretation and computational reuse are therefore still needed (Wise et al., 2019; Leipzig et al., 2021).

To address these limitations, we developed MAOMAO (Metadata-Aware Ontology for Multi-source Annotation Organization), an ontology-guided resource integrating peptide-toxicity information from public databases, literature-derived resources, and curated datasets. MAOMAO standardizes toxicity terminology, preserves source-level provenance, distinguishes uncertainty-related evidence states, and organizes annotations within a unified hierarchical framework. The resource also provides harmonized metadata, descriptor-based representations, protein language model embeddings, benchmark resources, and supporting documentation. These components establish a FAIR-oriented and reusable foundation for biological interpretation, computational toxicology, and predictive modeling of peptide toxicity.

## 2 Materials and methods

The workflow used to construct MAOMAO is summarized in Figure 1A. Toxicity-related information from heterogeneous sources was preprocessed, harmonized, mapped to a unified toxicity ontology, evaluated for annotation conflicts and evidence quality, enriched with standardized metadata, and consolidated into the final resource. To support dissemination, computational reuse, and long-term maintenance, MAOMAO was organized into a modular architecture comprising complementary resource layers (Figure 1B).

**FIGURE 1:**
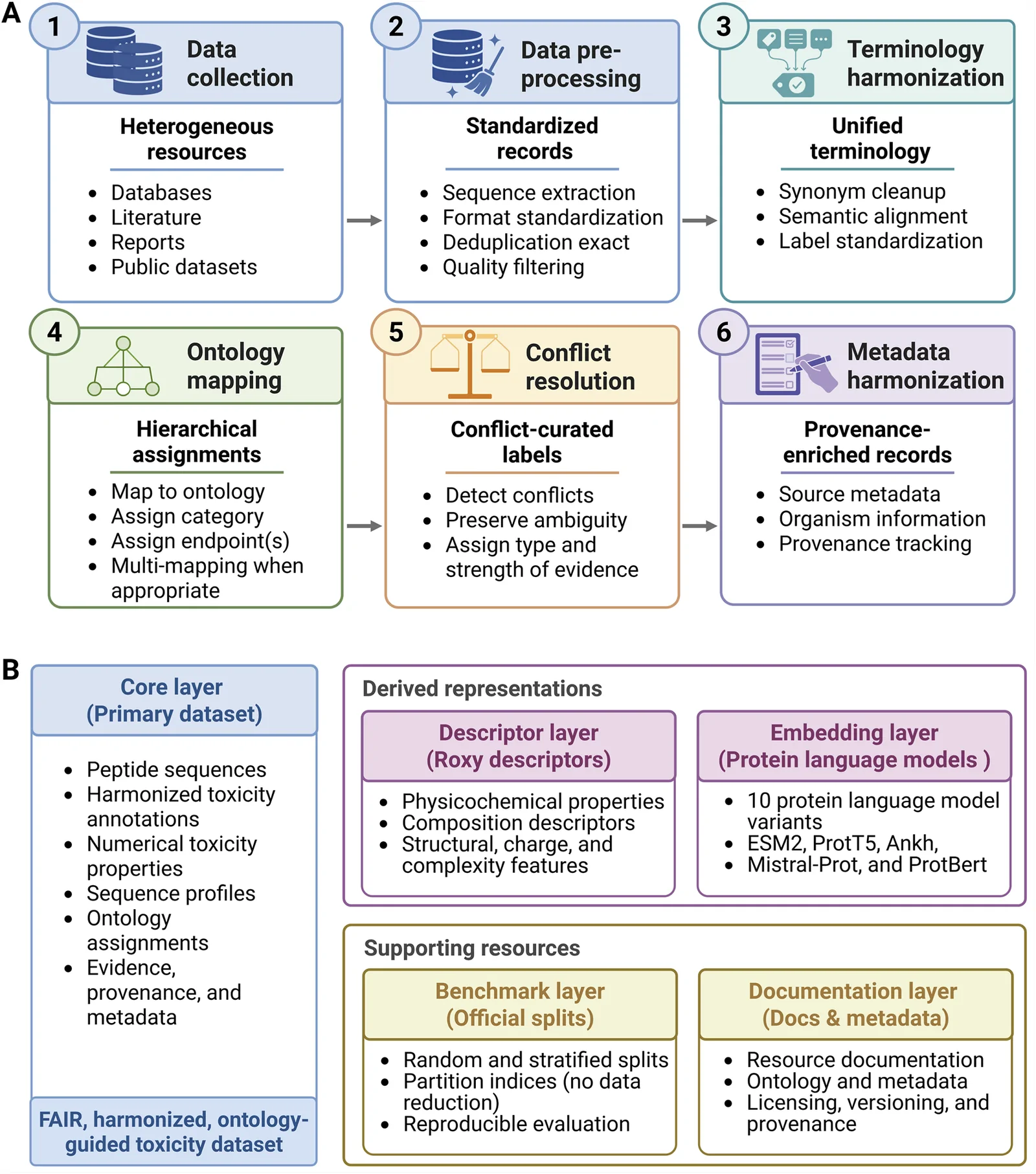
Overview of the MAOMAO construction workflow and resource architecture. **(A)** Ontology-guided harmonization workflow used to construct the resource. Toxicity-related information collected from public databases, literature-derived datasets, and curated resources undergoes preprocessing, terminology harmonization, ontology mapping, evidence integration, quantitative-property parsing, and metadata harmonization to generate a standardized and provenance-aware toxicity resource. **(B)** Modular architecture of MAOMAO. The resource is organized into complementary layers comprising harmonized toxicity annotations, numerical toxicity properties, sequence profiles, and metadata (Core Layer), sequence-derived descriptors (Descriptor Layer), protein language model embeddings (Embedding Layer), benchmark resources (Benchmark Layer), practical usage examples, and supporting documentation (Documentation Layer). Together, these components establish a FAIR-oriented and reusable resource for peptide toxicity research.

### 2.1 Data collection

MAOMAO was constructed by integrating toxicity-related information from publicly available databases, literature-derived resources, supplementary datasets, and manually curated repositories. Candidate resources were identified through a structured exploration of the peptide toxicity landscape using scientific search engines, bibliographic repositories, specialized peptide databases, review articles, and citation tracking (Asim et al., 2025; Mariano et al., 2017). Searches combined general terms, including *peptide toxicity*, *toxic peptides*, *toxic peptide databases*, and *toxic peptide predictive models*, with endpoint-specific queries for *hemolytic peptides*, *cytotoxic peptides*, *cytolytic peptides*, *neurotoxic peptides*, *embryotoxic peptides*, *ichthyotoxic peptides*, and *anti mammalian cells peptides*. Additional resources were identified from toxicity prediction tools, benchmark datasets, supplementary materials, associated repositories, and specialized peptide resources. Searches were conducted using Google Scholar and PubMed (Fiorini et al., 2017) between June 2025 and September 2026 and covered resources available through September 2026.

Candidate resources were evaluated according to predefined eligibility criteria, including relevance to at least one MAOMAO toxicity endpoint, availability of peptide sequences with corresponding categorical toxicity annotations or quantitative toxicity measurements, sufficient provenance information, and the possibility of programmatic or manual retrieval. Resources were excluded when the underlying dataset could not be retrieved through the reported publication, supplementary material, repository, or web server, or when the available material lacked sequences suitable for sequence-level integration. This included resources containing only numerical representations, peptide names, or functional annotations without the corresponding sequences. Candidate-source screening, eligibility decisions, integrated and excluded resources, and source-specific exclusion reasons are detailed in Supplementary Section S1.

Data acquisition combined direct database downloads, repository and supplementary-file retrieval, and manual extraction when required. Retrieved records were converted into a standardized intermediate representation, preserving peptide sequences, toxicity annotations, quantitative toxicity measurements when available, source identifiers, literature references, and available metadata. Source-level quality control verified the reported access route, the availability of primary sequences and interpretable toxicity information, and the associated publication or repository. Records lacking peptide sequences, containing toxicity information that could not be interpreted, or presenting irrecoverable formatting inconsistencies were excluded. Retained records were then forwarded to preprocessing, terminology harmonization, ontology mapping, metadata harmonization, and evidence integration.

### 2.2 Data preprocessing, terminology harmonization, and ontology mapping

Following data collection, records underwent standardized preprocessing to support integration across heterogeneous resources (Figure 1A). This stage included sequence validation, format standardization, annotation parsing, identifier normalization, and quality control. The final resource was restricted to peptides containing the 20 canonical amino acids and lengths between 5 and 70 residues (Xiao et al., 2025; Quiroz et al., 2021). Recoverable formatting inconsistencies were corrected, exact duplicates within each source were removed, and identical sequences reported by different sources were retained to preserve provenance and support evidence integration. Each normalized sequence was assigned a deterministic identifier generated by applying SHA-256 to the UTF-8 encoding of the uppercase, whitespace-free sequence. Quantitative HC50, LC50, LD50, and MHC observations were parsed while preserving their reported relation, value, unit, and provenance, and were linked to Core sequences through this identifier. Detailed sequence-processing, identifier-construction, and quantitative-property procedures are provided in Supplementary Section S1.

To prevent terminology inconsistencies during cross-source integration, toxicity-related annotations were examined for alternative spellings, synonyms, endpoint-specific descriptions, and source-dependent nomenclature referring to equivalent biological concepts. These variants were mapped to a standardized vocabulary while preserving their original biological meaning. For example, *haemolytic* was normalized to *hemolytic*, and *cytolytic* to *cytolysis*.

Harmonized annotations were mapped to the MAOMAO toxicity ontology, centered on the root category Toxic. The ontology comprises Cytotoxic, Neurotoxic, Embryotoxic, and Ichthyotoxic as primary classes, with Hemolytic, Cytolysis, and Anti mammalian cells represented as subclasses of Cytotoxic. Hemolytic denotes toxicity involving erythrocyte lysis, whereas Cytolysis captures cell lysis or membrane-disruptive activity without a specified cell type. Anti-mammalian-cell activity denotes damage to mammalian cells or reduced cell viability (Nygaard et al., 2025; Riss et al., 2016; Matsuzaki, 2009). The vocabulary was restricted to distinct toxicity concepts supported by retrievable sequence-level evidence. Complete endpoint definitions, mappings, and assignment rules are provided in Supplementary Section S2.

### 2.3 Conflict resolution and evidence management

Following data collection, harmonization, and ontology mapping, annotations associated with identical peptide sequences were aggregated across sources (Figure 1A). Because the same sequence could be reported with different toxicity annotations, an evidence-management framework was applied to classify each peptide–endpoint pair as positive, negative, ambiguous, unlabeled, or no information. Positive states represented direct or hierarchy-supported toxicity evidence, whereas negative states corresponded to direct endpoint-specific non-toxic evidence. Ambiguous states captured conflicting or unresolved annotations. Unlabeled states denoted sequences retained in endpoint-specific sources without an explicit class assignment, while no information indicated the absence of evidence for that endpoint.

Negative evidence was further classified as strong or weak according to the supporting information available in the original source. Strong negatives were explicitly supported by experimental evidence, whereas weak negatives originated from randomly selected negative sets or lacked confirmation of the absence of toxicity annotations. Evidence strength was retained in the metadata and provenance records, enabling downstream analyses to account for confidence, uncertainty, and conflicting evidence. Definitions, assignment criteria, and conflict-resolution rules are provided in Supplementary Section S2.

### 2.4 Metadata harmonization and provenance tracking

To support transparency, interoperability, and long-term reuse, source- and endpoint-level metadata were normalized into a unified schema. Harmonized fields included source identifiers, literature references, organism information, annotation provenance, retrieval dates, file formats, and version information when available. Missing values were explicitly preserved without inference. The schema also retained original source labels, ontology mappings, evidence categories, publication references, and resource-specific annotations, providing a standardized representation of the integrated biological and technical information.

Provenance was maintained by linking harmonized annotations to their source-specific processed datasets, endpoint-integration outputs, and metadata records. The sequence-level pivot provides the harmonized access layer, while provenance records, the ambiguity-support table, and the hierarchy-audit table document evidence aggregation, conflict resolution, and hierarchy-derived changes. These files allow each sequence–endpoint state to be traced to its supporting sources and processing decisions. Metadata fields, controlled vocabularies, and data types are detailed in Supplementary Section S3.

### 2.5 Resource architecture

To support reproducibility, computational reuse, and long-term maintenance, MAOMAO was organized into complementary functional layers (Figure 1B). This modular architecture separates harmonized biological data, computational representations, benchmark resources, and supporting documentation while preserving interoperability across the resource.

The Core Layer contains harmonized peptide sequences, toxicity annotations, ontology mappings, evidence classifications, provenance information, and metadata. It additionally provides numerical toxicity properties and sequence profiles. Descriptor-based representations were generated using Roxy (Medina‐Ortiz, 2026b), whereas embedding-based representations were obtained with Sylphy (Medina‐Ortiz, 2026c) using publicly available protein language models.

Benchmark resources were generated using partitioning strategies implemented through BioSieve (Medina‐Ortiz, 2026a). The Documentation Layer contains ontology specifications, metadata schemas, resource descriptions, licensing information, and versioning records. Supported representations, benchmark resources, resource organization, and file structures are detailed in Supplementary Section S4,5.

### 2.6 FAIR implementation

FAIR principles guided the design and construction of MAOMAO to support transparency, reproducibility, interoperability, and long-term sustainability. FAIR-oriented practices were incorporated across data collection, terminology harmonization, ontology development, evidence management, metadata integration, and resource organization.

Interoperability and reusability were supported through standardized toxicity terminology, ontology-guided endpoint mapping, harmonized metadata schemas, explicit evidence categorization, provenance tracking, and version-aware organization. These components enable heterogeneous toxicity annotations to be integrated while preserving the original information associated with each record.

Findability, accessibility, and computational reuse were supported by distributing resource components in machine-readable formats and using standardized identifiers linking sequences, annotations, metadata, ontology terms, and evidence categories. The sequence-derived SHA-256 identifiers provide a stable key for linking corresponding records across the Core, Descriptor, Embedding, Benchmark, and sequence-profile resources. Harmonization procedures, ontology definitions, evidence-management rules, metadata specifications, and generation workflows were also documented to support validation, updates, and downstream use. Resource availability, file organization, versioning, and supporting documentation are detailed in Supplementary Section S5.

## 3 Resource description

### 3.1 Dataset overview

An overview of MAOMAO is presented in Figure 2A. The resource integrates toxicity-related information from 56 publicly available databases, literature-derived resources, supplementary datasets, and manually curated repositories. Of 1,372,190 non-deduplicated source-level records initially collected, 898,637 were retained after source-specific preprocessing, representing a reduction of approximately 34.5%. Removed records included duplicates, incomplete entries, formatting inconsistencies, and annotations that did not satisfy the inclusion criteria.

**FIGURE 2:**
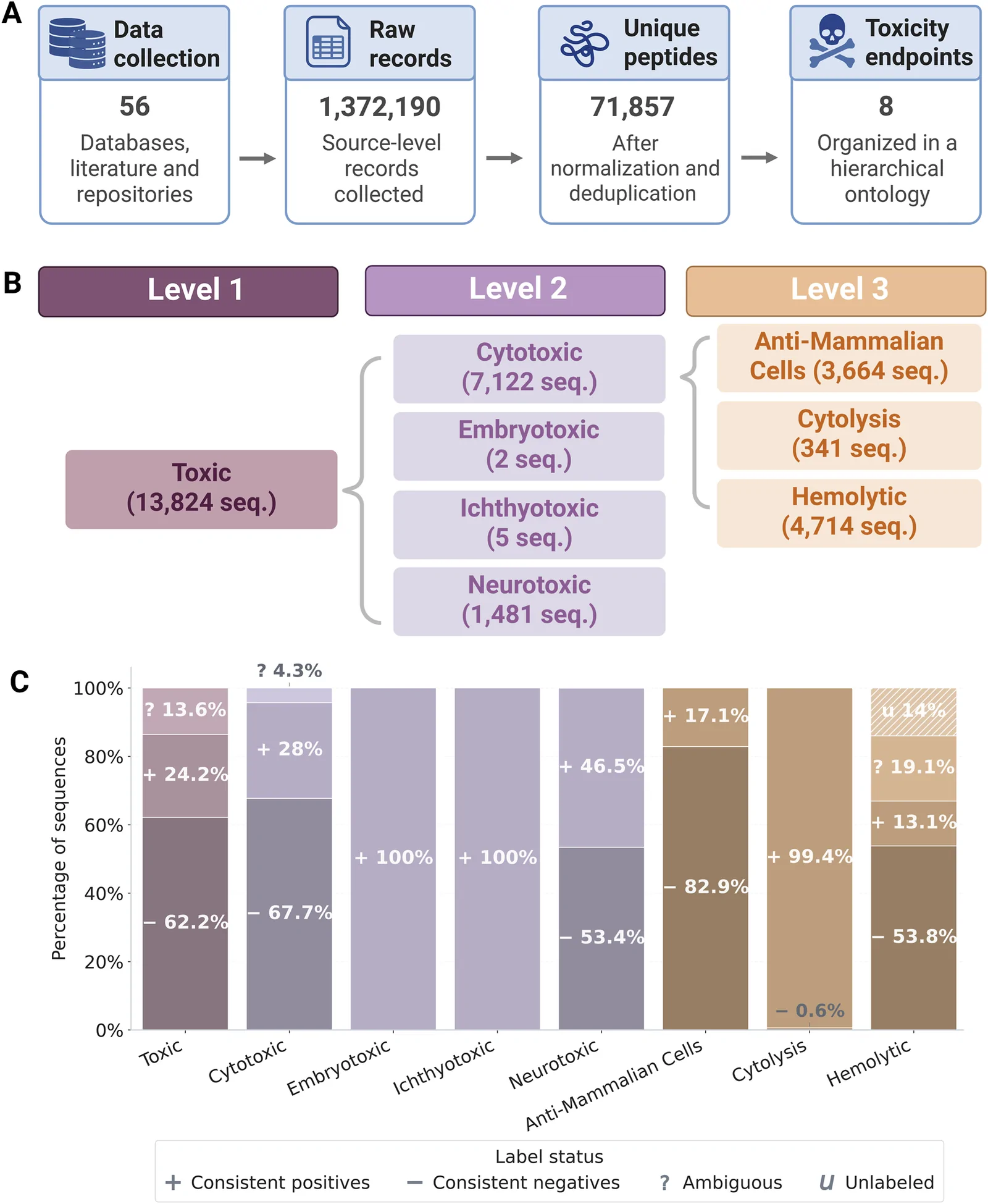
Organization, composition, and coverage of the MAOMAO resource. **(A)** General overview of the resource, including the number of integrated sources, source-level records, unique peptide sequences, and toxicity-related endpoints. **(B)** Hierarchical organization of the toxicity endpoints included in MAOMAO. Numbers indicate the number of unique peptide sequences with positive evidence for each endpoint after hierarchy application. **(C)** Composition of sequence–endpoint states across evidence categories, including positive, negative, ambiguous, and unlabeled states, illustrating the distribution of available evidence for each toxicity endpoint.

Following terminology harmonization, ontology mapping, cross-source evidence aggregation, state resolution, and quality control, the final release comprised 71,857 unique peptide sequences across eight toxicity-related endpoints. These were organized into 574,856 sequence–endpoint combinations, together with standardized metadata and provenance records linking annotations, ontology assignments, evidence classifications, and source-specific information.

Publication references, repository or server information, download dates, file formats, source types, and resource behavior were documented for all 56 sources. Annotation content and last-update dates were available for 55 sources (98.21%), whereas explicit licensing information was available for 15 sources (26.79%). Missing metadata were preserved without inference. These components provide a harmonized resource for organizing, disseminating, and computationally analyzing peptide toxicity information.

### 3.2 Toxicity ontology and endpoint organization

The ontology-guided organization of toxicity annotations is presented in Figure 2B. The root category Toxic contains 57,080 available peptide–endpoint states, including 13,824 positives, and comprises four primary classes, namely, Cytotoxic (25,454 available states; 7,122 positive), Neurotoxic (3,183; 1,481 positive), Embryotoxic (2 positive), and Ichthyotoxic (5 positive). The Cytotoxic branch is further divided into Hemolytic (35,960 available states; 4,714 positive), Cytolysis (343; 341 positive), and Anti mammalian cells (21,393 available states; 3,664 positive), preserving more specific biological contexts. These categories define the endpoint structure used throughout MAOMAO.

Evidence-state distributions are summarized in Figure 2C and Supplementary Section S10. Across MAOMAO, 31,153 states were positive, 91,506 negative, 15,717 ambiguous, and 5,044 unlabeled. Because only positive annotations were propagated through the hierarchy, all other states remained endpoint-specific, and no-information states were excluded from the percentages shown in Figure 2C. Toxic contained 13,824 positives, whereas Hemolytic had the highest proportion of ambiguous states (19.05%) and was the only endpoint with unlabeled states, representing 14.03% of its available annotations.

Across the seven non-root endpoints, 7,331 of the 9,421 peptides with at least one positive annotation were assigned to multiple toxicity categories. This corresponds to 77.82% of positive peptides across these endpoints and 10.2% of all MAOMAO sequences. Percentages were calculated relative to the positive sequences assigned to each row endpoint and should therefore be interpreted directionally. The largest overlap occurred between Cytotoxic and Hemolytic, with 4,128 shared sequences, representing 57.96% of cytotoxic-positive and 87.57% of hemolytic-positive sequences. Complete pairwise counts and directional percentages are provided in Supplementary Section S2.

### 3.3 Resource contents

MAOMAO distributes harmonized annotations, numerical representations, benchmark resources, metadata, ontology definitions, and documentation across the functional layers shown in Figure 1B.

The Core Layer contains 574,856 harmonized peptide–endpoint combinations corresponding to 71,857 unique sequences, together with ontology assignments, evidence classifications, provenance, and standardized metadata. It additionally provides 169 HC50, LC50, LD50, and MHC measurement records. Most were linked to the Core sequence universe, while six LD50 records remained unmatched because their sequences exceeded the 70-residue limit. One profile per Core sequence summarizes its activity annotations, supporting evidence, ontology effects, toxicity targets, selected descriptors, and available numerical toxicity properties. The Descriptor Layer provides 41 features capturing sequence composition and physicochemical properties.

The Embedding Layer includes 10 protein-language-model embedding matrices and a one-hot representation, all covering the complete set of 71,857 MAOMAO sequences.

The Benchmark Layer contains 300 endpoint–seed–strategy configurations generated through random and stratified five-fold partitioning of five eligible endpoints (Toxic, Cytotoxic, Hemolytic, Neurotoxic, and Anti mammalian cells) using 30 random seeds. These correspond to 1,500 data partitions and, across the 11 numerical representations, 3,300 representation-specific configurations and 16,500 fold instances. Associated parameters, reports, summaries, and execution records are also provided.

The Documentation Layer includes ontology definitions, metadata schemas, resource descriptions, versioning records, and licensing information. Files are distributed in standard formats, including CSV, JSON, and YAML, with detailed component descriptions and inventories provided in Supplementary Section S4,5.

### 3.4 Practical resource reuse and applications

To facilitate direct reuse of the distributed assets, MAOMAO provides five executable notebooks that operate on the released files rather than reconstructing the resource from the original sources. The introductory workflow covers resource access, file validation, navigation of the Core Layer, and linkage of annotations, numerical representations, and benchmark partitions through stable sequence identifiers. Complementary notebooks support descriptive characterization of endpoint states, class balance, co-occurrence, sequence lengths, ontology consistency, and representation coverage, as well as retrieval and querying of individual sequence profiles. Generated tables and figures are stored separately from the released resource files.

Two additional notebooks illustrate downstream machine-learning reuse. The neurotoxicity classification example joins a released train–validation–test partition with an existing protein language model representation and evaluates logistic regression, a linear support vector machine, and random forest models using fixed settings. The quantitative regression example shows how MAOMAO’s numerical toxicity measurements can be combined with a released sequence representation in a simple regression analysis. These workflows are intended to demonstrate resource interoperability and reproducible reuse rather than establish state-of-the-art predictors or replace the released benchmark design. Detailed descriptions of the demonstration notebooks, execution requirements, and generated outputs are provided in Supplementary Section S6.

## 4 Data value and reuse potential

MAOMAO was designed as a reusable resource for peptide toxicity research, providing a harmonized collection of toxicity annotations, ontology mappings, evidence classifications, metadata records, numerical representations, and benchmark resources. By integrating heterogeneous toxicity information into a unified framework, the resource reduces challenges in data acquisition, terminology inconsistencies, annotation ambiguity, and dataset construction, enabling researchers to focus on downstream biological and computational analyses.

MAOMAO supports predictive toxicology. The resource provides standardized toxicity endpoints, evidence-aware annotations, and benchmark partitions that can be used to develop, compare, and evaluate machine learning models for toxicity prediction. The availability of descriptor-based representations, protein language model embeddings, and predefined dataset partitions facilitates reproducible benchmarking and comparative evaluation of computational approaches. Numerical toxicity properties extend this utility to exploratory regression and quantitative activity analyses, while sequence profiles enable sequence-level inspection of annotations, supporting evidence, ontology effects, toxicity targets, and available measurements.

Beyond supervised learning, MAOMAO may support positive–unlabeled learning, weak supervision, uncertainty-aware modeling, multi-task and multi-endpoint learning, and explainability analyses. The explicit preservation of annotation provenance, evidence categories, and ontology mappings allows computational approaches to account for annotation uncertainty and supports evidence-aware interpretation and model transparency. The harmonized annotation structure also enables toxicity landscape characterization, endpoint co-occurrence analyses, toxicity-aware peptide discovery, and safety-oriented peptide design.

Despite this reuse potential, endpoint coverage depends on the availability of retrievable sequence-level evidence across the curated sources, resulting in class imbalance and limited representation of some endpoints, particularly Cytolysis, Embryotoxic, and Ichthyotoxic. Metadata completeness and evidence strength also vary across sources, and this heterogeneity may affect downstream analyses despite the preservation of provenance, conflicting annotations, and evidence categories. Finally, the predefined benchmark partitions rely on random and stratified splitting without homology- or similarity-based control and should therefore not be interpreted as leakage-controlled evaluation datasets.

Future releases may address these limitations through the incorporation of newly available toxicity datasets, expanded endpoint and numerical-property coverage, metadata enrichment, updated numerical representations, and similarity-aware benchmark partitions. The modular architecture and deterministic sequence identifiers provide a traceable basis for extending MAOMAO across resource versions.

## Data Availability

The complete, versioned MAOMAO resource is archived on Zenodo: https://zenodo.org/communities/kren-ai-lab, DOI: https://doi.org/10.5281/zenodo.22261793. Code, workflows, notebooks, and environment specifications are available at https://github.com/kren-ai-lab/maomao under the MIT License.
