# Supplementary material for: MAOMAO: an ontology-guided FAIR resource for harmonized peptide toxicity data
Source: Front Pharmacol. 2026 Oct 16;17:1952924. doi: 10.3389/fphar.2026.1952924 (PMC13719554; doi:10.3389/fphar.2026.1952924)
Supplement: Supplementary file 1 [file DataSheet1.pdf]

Supplementary Information: MAOMAO: An Ontology-Guided FAIR Resource for Harmonized Peptide Toxicity Data

Nicole Soto-Garcia[1], Julián García-Vinuesa[1], Roberto Uribe-Paredes[1], Leandro Murgas[1], Karen Oróstica[2], Jorge González-Puelma[3,4], Marcelo Navarrete[3,4], Frederic Cadet[5], and David Medina-Ortiz[1,*]

[1]Departamento de Ingeniería en Computación, Universidad de Magallanes, Punta Arenas, Chile.
[2]Data Science Institute, Universidad del Desarrollo, Santiago, Chile.
[3]Centro Asistencial Docente e Investigación, Universidad de Magallanes, Punta Arenas, Chile.
[4]Escuela de Medicina, Universidad de Magallanes, Punta Arenas, Chile.
[5]PEACCEL, AI for Biologics, Paris, France.
* Corresponding author: David Medina-Ortiz (david.medina@umag.cl)

Supplementary contents

S1 Dataset construction and source curation **2**
S1.1 Bibliographic search and source identification . . . 2
S1.2 Inclusion and exclusion criteria . . . 3
S1.3 Source screening and selection outcome . . . 4
S1.4 Excluded candidate sources . . . 4
S1.5 Source datasets used for peptide toxicity data curation . . . 5
S1.6 Source-level parsing and sequence quality control . . . 6

S2 Terminology harmonization and toxicity ontology **8**
S2.1 Endpoint definitions used for ontology-guided harmonization . . . 8
S2.2 Evidence-state definitions and conflict resolution . . . 9
S2.3 Hierarchy propagation rules and audit statistics . . . 9
S2.4 Final endpoint-state composition . . . 10
S2.5 Positive endpoint co-annotation analysis . . . 10

S3 Metadata schema and provenance fields **11**
S3.1 Metadata schema . . . 12
S3.2 Source metadata coverage . . . 14
S3.3 Source access and licensing audit . . . 14

S4 Computational resource generation and file organization **16**

S4.1 Descriptor Layer generation . . . 16
S4.2 Numerical representation extraction . . . 17
S4.3 Supported protein language model embeddings . . . 18
S4.4 One-hot baseline representation . . . 18
S4.5 Benchmark partition generation . . . 18
S4.6 File organization . . . 20

S5 Resource contents and file summaries **22**
S5.1 Core Layer outputs . . . 22
S5.2 Descriptor Layer outputs . . . 23
S5.3 Embedding Layer outputs . . . 23
S5.4 Benchmark partition resources . . . 23
S5.5 Release-level organization . . . 24
S5.6 Release, versioning, and licensing . . . 25

S6 Demonstration notebooks and worked examples **25**
S6.1 Notebook scope and organization . . . 25
S6.2 Illustrative resource characterization and neurotoxicity classification . . . 25
S6.3 Illustrative quantitative activity regression . . . 27
S6.4 Sequence-profile exploration and output separation . . . 27

S1 Dataset construction and source curation

This section outlines how the MAOMAO resource was constructed and refined. It covers the identification, selection, extraction, and harmonization of source datasets, and the procedures applied to resolve duplicate sequences and conflicting annotations prior to further processing.

S1.1 Bibliographic search and source identification

Candidate sources were identified through a structured search focused on publicly available resources containing sequence-level information associated with peptide toxicity. The search was conducted between June 2025 and September 2026 using Google Scholar and PubMed, and covered resources available through September 2026. The objective was to identify databases, published datasets, supplementary materials, repositories, and datasets associated with peptide toxicity prediction studies that could contribute sequences and annotations to at least one of the toxicity endpoints represented in MAOMAO. Search terms combined general concepts related to peptide toxicity and toxicity-oriented data resources. Searches were not restricted by publication year, and resources available up to September 2026 were considered. The bibliographic search was complemented by inspection of review articles, specialized peptide databases, publisher pages, article supplementary materials, associated repositories, and resources linked to peptide toxicity prediction tools and benchmark datasets. Citation tracking was also performed using references and dataset descriptions from relevant studies to identify earlier datasets, database versions, and primary sources that had been reused, reformatted, or expanded in subsequent publications. Table **S1** summarizes the main search dimensions used during candidate-source identification. Priority was given to resources that provided primary peptide sequences together with toxicity annotations or labels that could be interpreted and mapped to the MAOMAO terminology.

Supplementary Table S1: Search strategy used to identify candidate sources for MAOMAO construction.

Search dimension	Terms or sources	Purpose
Bibliographic search	Google Scholar and PubMed	Identify publications, databases, prediction studies, and publicly available datasets containing peptide-toxicity information.
General toxicity concepts	peptide toxicity, toxic peptides, toxic peptide databases, and toxic peptide predictive models	Identify general toxicity resources and datasets used for peptide-toxicity research or predictive modeling.
Endpoint-specific search	hemolytic peptides, cytotoxic peptides, cytolytic peptides, neurotoxic peptides, embryotoxic peptides, ichthyotoxic peptides, and anti mammalian cells peptides	Identify resources focused on the specific toxicity endpoints represented in MAOMAO.
Prediction and benchmark resources	Peptide-toxicity prediction tools, model publications, and benchmark datasets	Identify sequence datasets used to train, validate, or compare peptide-toxicity prediction models.
Specialized resource inspection	Peptide databases, review articles, publisher pages, and manually curated repositories	Identify candidate resources not recovered directly through the bibliographic search.
Data availability screening	Article supplementary materials, database downloads, associated repositories, web servers, and linked code or data repositories	Determine whether primary peptide sequences and their corresponding toxicity annotations could be retrieved.
Citation tracking	Reference lists, dataset descriptions, and cited sources from relevant peptide-toxicity studies	Identify earlier datasets, original data sources, database versions, and resources reused or expanded in subsequent publications.

Candidate resources were initially recorded independently for each toxicity endpoint under which they were identified. A source associated with more than one endpoint could therefore appear in multiple endpoint-specific searches. For the calculation of global source-selection counts, source names were standardized and repeated resources were counted only once. Distinct database versions were retained as separate sources when they represented independently released or curated datasets.

When a publication, database, or repository provided multiple files, supplementary tables, dataset partitions, or endpoint-specific subsets, these components were treated as part of the same source rather than as independent sources. All retrieved files were retained during the initial compilation step to preserve their provenance and to support subsequent source-level and record-level consistency assessment. Source name, publication or repository, access route, retrieval date, file format, toxicity endpoint, sequence counts, and available annotation information were recorded before preprocessing, harmonization, and cross-source integration.

S1.2 Inclusion and exclusion criteria

Candidate sources were considered eligible for sequence-level curation when they met all of the following criteria:

(i) the source contained information relevant to at least one toxicity endpoint represented in MAOMAO;

(ii) peptide sequences were available from the database, publication, supplementary material, web server, or an associated repository;

(iii) the source provided toxicity annotations, labels, activity descriptions, or dataset-class definitions that could be interpreted and mapped to the MAOMAO terminology;

(iv) the underlying data could be retrieved through a documented access route using programmatic or manual procedures.

(v) sufficient provenance information was available to associate the retrieved records with the corresponding database, publication, dataset, repository, or other documented source.

Candidate sources were excluded when the underlying dataset could not be retrieved through the reported publication, supplementary material, database, repository, or web server. Sources were also excluded when the accessible material did not provide the peptide sequences required for sequence-level integration. This category included resources containing only numerical representations, peptide names, Gene Ontology annotations, or other functional metadata without the corresponding amino-acid sequences.

Source-level exclusion was distinguished from record-level filtering. A source was considered included when at least part of its sequence-level dataset met the eligibility criteria and could be retrieved. After source inclusion, individual records could still be removed during preprocessing when they lacked peptide sequences, contained incomplete or uninterpretable toxicity annotations, presented irrecoverable formatting inconsistencies, or did not satisfy the sequence-quality criteria applied during harmonization.

S1.3 Source screening and selection outcome

After standardization of source names, 77 unique candidate sources were identified and assessed for eligibility. Of these, 56 met the inclusion criteria and were retained for sequence-level curation and integration, whereas 21 were excluded. Among the excluded sources, 17 could not be incorporated because the corresponding datasets were either unavailable through the reported access routes or no data-access route was provided. The remaining 4 did not provide usable peptide sequences: 2 contained only numerical representations, 1 listed peptide names without the corresponding sequences, and 1 reported peptide names and Gene Ontology annotations without the corresponding sequences.

Table **S2** summarizes the source-identification and eligibility-assessment process, including the number of unique candidate sources evaluated, the principal reasons for exclusion, and the number of sources retained for MAOMAO construction.

Supplementary Table S2: Summary of candidate-source identification, eligibility assessment, and selection for MAOMAO construction. Counts correspond to unique sources after source-name standardization.

Selection stage	Sources	Description
Candidate sources identified	77	Unique candidate resources identified through bibliographic searches, resource inspection, and citation tracking.
Candidate sources evaluated	77	Resources assessed according to toxicity relevance, sequence availability, annotation accessibility, data retrievability, and provenance.
Sources excluded	21	Resources that did not satisfy the source-level eligibility criteria.
Underlying data unavailable	17	The corresponding dataset could not be retrieved through the reported publication, supplementary material, database, repository, or web server, or no data-access route was provided.
No usable primary sequences	4	The accessible resources provided no sequences, only numerical representations, or names and functional annotations without the corresponding amino-acid sequences.
Sources integrated	56	Eligible resources retained for sequence extraction, preprocessing, harmonization, ontology mapping, and evidence integration.

These counts represent unique sources rather than source–endpoint associations, because a single resource could contribute information to more than one toxicity endpoint. The excluded candidate sources and their source-specific reasons for exclusion are reported in Table **S3**, whereas the endpoint coverage, retained sequence counts, and annotation categories of the 56 integrated sources are summarized in Table **S4**.

S1.4 Excluded candidate sources

Not all candidate resources identified during source discovery could be incorporated into MAOMAO. Table **S3** lists the 21 sources that did not meet the source-level eligibility criteria and summarizes the corresponding reasons for exclusion.

Supplementary Table S3: Candidate peptide-toxicity sources excluded during source-level eligibility assessment, grouped according to the primary reason for exclusion.

Reason for exclusion	Sources	Candidate sources
Dataset unavailable or no usable data-access route	17	Toropov et al. [Toropov et al., 2025]; AMP-D3 (HemolyticPredictor) [Ouyang et al., 2025]; Kumar et al. [Kumar et al., 2025]; AmpLyze [Qiu et al., 2025]; Castillo-Mendieta et al. [Castillo-Mendieta et al., 2024b]; Ansari et al. [Ansari and White, 2023a]; MoFormer [Wang et al., 2024a]; MQSS model [Castillo-Mendieta et al., 2024a]; Ansari et al. (2023) [Ansari and White, 2023b]; HemoPred [Win et al., 2017]; Wan et al. [Wan et al., 2023]; PEP-PREDNa+ [Herrera-Bravo et al., 2022]; Neu_LR [Zhu et al., 2021]; M3-CAD [Wang et al., 2024b]; ToxMVA [Shi et al., 2022]; ATSE [Wei et al., 2021]; and Khabbaz et al. [Khabbaz et al., 2021].
Only numerical representations available	2	AIPAMPDS (HemoRisk-Estimator) [Li et al., 2025] and ToxDL [Pan et al., 2020].
Only peptide names and/or functional annotations available	2	PlantPepDB [Das et al., 2020] and NNTox [Jain and Kihara, 2019].

For PlantPepDB, the accessible material provided only peptide names and did not include the corresponding sequences required for sequence-level integration. NNTox provided peptide names and Gene Ontology annotations, but not the corresponding amino-acid sequences.

These exclusions were applied at the source level before record extraction and were therefore distinct from the subsequent record-level filtering of entries with missing sequences, uninterpretable toxicity annotations, or irrecoverable formatting inconsistencies within the 56 eligible sources.

S1.5 Source datasets used for peptide toxicity data curation

This subsection presents the source datasets that were kept for building the peptide toxicity dataset following the bibliographic search and eligibility screening. Table **S4** shows, for each source, the toxicity effects covered, the number of records retained after source-specific processing, and the distribution of positive, negative, unlabeled, and other entries reported or derived from each dataset. These figures reflect source-level data before cross-source consolidation, global terminology harmonization, evidence-state resolution, and hierarchy application.

Supplementary Table S4: Overview of source datasets used for peptide toxicity data curation. Retained values are non-deduplicated source-level record counts after source-specific processing. Annotation-category counts describe the retained records before cross-source consolidation, evidence-state resolution, and hierarchy application. The *Other* column denotes source-specific records outside the positive, negative, and unlabeled categories, such as regression records. Across the 56 source metadata records, 1,372,190 raw records were reported and 898,637 records were retained.

Source	Toxicity endpoint(s)	Retained records	Positive	Negative	Unlabeled	Other
Abdelbaky et al. [Abdelbaky et al., 2024]	Hemolytic; toxic	5851	1983	3868	0	0
Almotairi et al. [Almotairi et al., 2024]	Hemolytic; toxic	5400	1907	3493	0	0
AMPDB v1 [Mondal et al., 2023]	Toxic; cytolytic; cytotoxic; hemolytic; ichthyotoxic; other toxicity-related effects	5230	5230	0	0	0
AMPDeep [Salem et al., 2022]	Hemolytic; toxic	16191	9449	6742	0	0
Bhatnagar et al. [Bhatnagar et al., 2024]	Hemolytic; toxic	756	167	589	0	0
BIOPEP-UWM [Minkiewicz et al., 2019]	Hemolytic; toxic; cytotoxic; embryotoxic	89	89	0	0	0
BiToxNet [Wang et al., 2026]	Neurotoxic; toxic	7780	2399	5381	0	0
CAPTP [Jiao et al., 2024]	Toxic	7513	2138	5375	0	0
CICERON [Bizzotto et al., 2024]	Hemolytic; toxic; embryotoxic; cytotoxic	82	82	0	0	0
ConsAMPHemo [Xie et al., 2025]	Hemolytic; toxic	5841	2246	3595	0	0
csm-toxin [Morozov et al., 2023]	Toxic	221634	7392	214242	0	0
DRAMP [Ma et al., 2025]	Toxic; hemolytic; cytolytic; cytotoxic	3225	530	2695	0	0

Continued on next page

Supplementary Table S4: Overview of source datasets used for peptide toxicity data curation. Continued.

Source	Toxicity endpoint(s)	Retained records	Positive	Negative	Unlabeled	Other
HAPPENN [Timmons and Hewage, 2020]	Hemolytic; toxic	3532	1456	2076	0	0
HemoDL [Yang and Xu, 2024]	Hemolytic; toxic	5437	2369	3068	0	0
HemoFuse [Zhao et al., 2024]	Hemolytic; toxic	5437	2369	3068	0	0
Hemolytic-Pred [Perveen et al., 2023]	Hemolytic; toxic	1235	0	0	1235	0
Hemolytik [Gautam et al., 2014]	Hemolytic; toxic	2471	2471	0	0	0
hemolytik 2.0 [Singh et al., 2025]	Hemolytic; toxic	4960	4960	0	0	0
hemonet [Yaseen et al., 2021]	Hemolytic; toxic	3308	1422	1851	35	0
HemoPI [Chaudhary et al., 2016]	Hemolytic; toxic	2191	910	1281	0	0
HemoPI2.0 [Rathore et al., 2025b]	Hemolytic; toxic	1926	891	1035	0	0
HEPAD [Chen et al., 2025]	Hemolytic; toxic	2996	910	2086	0	0
HLPpred-Fuse [Hasan et al., 2020]	Hemolytic; toxic	4365	1360	3005	0	0
HMAMP-main [Wang et al., 2025a]	Hemolytic; toxic	1656	552	552	552	0
HyPepTox-Fuse [Tran et al., 2025]	Toxic	11036	5518	5518	0	0
iAMPCN [Xu et al., 2023]	Anti mammalian cells; Cytotoxic; hemolytic; toxic	84381	6343	78038	0	0
Karasev et al. [Karasev et al., 2024]	Hemolytic; toxic	1740	0	0	944	796
MultiPep [Grønning et al., 2021]	Hemolytic; toxic; embryotoxic; other toxicity-related effects	6400	6400	0	0	0
Multitox [Sharma et al., 2025]	Neurotoxic; hemolytic; cytotoxic; toxic	1898	1898	0	0	0
NTXpred [Saha and Raghava, 2007]	Neurotoxic; toxic	916	916	0	0	0
NTXpred2 [Rathore et al., 2025a]	Neurotoxic; toxic	3300	1649	1651	0	0
Pep-Lab_db [Terziyski et al., 2023]	Toxic	249	249	0	0	0
peptidereactor [Spänig et al., 2021]	Hemolytic; toxic	1104	522	582	0	0
Peptipedia 2.0 [Cabas-Mora et al., 2024]	Toxic; neurotoxic; hemolytic; cytotoxic	22455	22455	0	0	0
PeptiTox [Wang et al., 2025b]	Toxic	3864	1932	1932	0	0
Plisson et al. [Plisson et al., 2020]	Hemolytic; toxic	9625	1076	1275	7274	0
PLPTP [Gao et al., 2025]	Toxic	7513	2138	5375	0	0
ProToxin [Yang et al., 2025]	Toxic	252536	6954	244829	753	0
QSVM-PEPTIDE [Zhuang et al., 2024]	Hemolytic; toxic	2195	911	1284	0	0
SATPdb [Singh et al., 2016]	Toxic	4130	4130	0	0	0
StrucToxNet [Jiao et al., 2025]	Toxic	9544	2491	7053	0	0
tAMPer [Ebrahimikondori et al., 2024]	Toxic	5708	1790	3918	0	0
ToxDL 2.0 [Zhu et al., 2025]	Toxic	11434	4593	6841	0	0
ToxGIN [Yu et al., 2024]	Toxic	4410	2205	2205	0	0
ToxIBTL [Wei et al., 2022]	Toxic	14092	5698	8394	0	0
ToxinPred [Gupta et al., 2013]	Toxic	19391	2084	17307	0	0
ToxinPred 2.0 [Sharma et al., 2022]	Toxic	35242	8233	27009	0	0
ToxinPred 3.0 [Rathore et al., 2023]	Toxic	11036	5518	5518	0	0
ToxiPep [Guan et al., 2025]	Toxic	9889	4938	4951	0	0
ToxMSRC [Zhang et al., 2025]	Toxic	7513	7513	0	0	0
ToxTeller [Wang and Sung, 2024]	Toxic	4329	2078	2251	0	0
TPpred-LE [Lv et al., 2023]	Toxic	2345	2345	0	0	0
UniDL4BioPep [Du et al., 2023]	Toxic	3864	1932	1932	0	0
Zhao et al. [Zhao et al., 2021]	Hemolytic; toxic	2186	1093	1093	0	0
Hemolytik2.0_2026 [Singh et al., 2026]	Hemolytic; toxic	2419	1910	509	0	0
PepNet [Han et al., 2024]	Anti mammalian cells; toxic	22787	3681	19106	0	0
**Total**	—	**898,637**	**174,475**	**712,573**	**10,793**	**796**

Across all source-level data records, 1,372,190 raw records were reported and 898,637 were retained after source-specific processing, corresponding to an overall reduction of approximately 34.5%. These values represent non-deduplicated source-level totals.

S1.6 Source-level parsing and sequence quality control

Following source selection, records obtained from each eligible resource were parsed and converted into a standardized sequence-level representation. Peptide sequences, source-specific toxicity annotations, quantitative toxicity measurements, source identifiers, literature or repository references, and available metadata were preserved whenever possible. Records were excluded from sequence-level integration when the peptide sequence was missing or when irrecoverable formatting inconsistencies prevented conversion into a valid sequence-associated record. Records contributing qualitative toxicity information were additionally required to contain an annotation, label, activity description, or class definition that could be interpreted for sub-

sequent harmonization and mapping to the MAOMAO terminology. Quantitative toxicity records were retained when the corresponding measurement type and numerical information could be parsed without unsupported interpretation.

Sequence-level quality control was subsequently applied using predefined residue-composition and length criteria. Peptide sequences were retained when their length ranged from 5 to 70 amino-acid residues, inclusive, and every sequence character belonged to the canonical amino-acid vocabulary: `A, C, D, E, F, G, H, I, K, L, M, N, P, Q, R, S, T, V, W, Y`. Sequences containing at least one residue outside this vocabulary, as well as sequences shorter than 5 or longer than 70 residues, were excluded from downstream integration.

Exact duplicate records occurring within the same source were removed during source-level processing. In contrast, identical peptide sequences reported by different sources were retained together with their source-specific annotations and provenance information. This distinction prevented repeated records within a source from artificially increasing its contribution while preserving evidence reported by different resources.

For quantitative toxicity records, measurement information was parsed separately from qualitative evidence-state resolution. When available, the measurement type, comparison relation, numerical value, associated error, measurement unit, original activity label, and source provenance were preserved. Multiple distinct measurements associated with the same peptide were retained rather than interpreted as conflicting qualitative evidence.

Conflicting qualitative annotations were handled during evidence integration. Peptide–endpoint pairs with conflicting or otherwise unresolved evidence across sources were classified as *ambiguous*, while preserving the provenance of the contributing sources.

The quality-control configuration and its outcomes were documented in the corresponding metadata records. Source-level metadata included the numbers of raw, retained, erroneous, and modified sequences. Endpoint-level metadata recorded whether the canonical-residue and length filters were applied, the minimum and maximum accepted sequence lengths, the number of sequences before and after each filter, sequence-length distribution statistics, and the final number of sequences retained for each toxicity endpoint. For sources containing quantitative toxicity information, measurement type and unit information were additionally retained with the corresponding sequence-level records and provenance. The record-processing, sequence-quality, and annotation-integration rules applied during MAOMAO construction are summarized in Table **S5**.

Supplementary Table S5: Record-processing, sequence-level quality-control, and annotation-integration rules applied during MAOMAO construction.

**Processing case**	**Rule applied**	**Treatment or interpretation**
Missing peptide sequence	No primary amino-acid sequence was available for the record.	The record was excluded from sequence-level integration.
Missing or uninterpretable toxicity annotation	The annotation, label, activity description, or class definition could not be interpreted for subsequent terminology harmonization.	The record was excluded from qualitative toxicity-annotation integration.
Quantitative toxicity measurement	A toxicity measurement could be parsed into a measurement type and numerical value, together with the comparison relation, error, and unit when available.	The measurement was retained with the corresponding peptide sequence and source provenance and was handled separately from qualitative evidence-state resolution.
Multiple quantitative measurements	More than one distinct quantitative measurement was associated with the same peptide sequence.	Distinct measurements were preserved rather than converted into an ambiguous qualitative evidence state.
Recoverable formatting inconsistency	Formatting differences could be corrected without changing the biological meaning of the sequence or annotation.	The record was standardized and retained.
Irrecoverable formatting inconsistency	The record could not be converted into a valid sequence–annotation entry without unsupported interpretation.	The record was excluded during source-level processing.
Non-canonical amino-acid residue	At least one character was not included in the canonical vocabulary `A, C, D, E, F, G, H, I, K, L, M, N, P, Q, R, S, T, V, W, Y`.	The sequence was excluded during canonical-residue filtering.
Sequence outside the accepted length range	The sequence contained fewer than 5 or more than 70 amino-acid residues.	The sequence was excluded during length filtering.
Sequence passing both filters	The sequence contained only canonical amino-acid residues and had a length between 5 and 70 residues, inclusive.	The sequence was retained for downstream harmonization and integration.

Continued on next page

Supplementary Table S5: Record-processing, sequence-level quality-control, and annotation-integration rules applied during MAOMAO construction. Continued.

Processing case	Rule applied	Treatment or interpretation
Exact duplicate within the same source	The same sequence and source-specific annotation record occurred more than once within a source.	Duplicate records were removed during source-level processing.
Identical sequence across different sources	The same peptide sequence was reported by more than one source.	The sequence was retained together with all source-specific annotations and provenance records.
Conflicting annotations across sources	The same peptide–endpoint pair was supported by both positive and negative evidence across different sources.	The integrated annotation was classified as *ambiguous*.

S2 Terminology harmonization and toxicity ontology

S2.1 Endpoint definitions used for ontology-guided harmonization

The MAOMAO toxicity ontology was developed as an operational framework for terminology harmonization and annotation integration rather than as an exhaustive classification of toxicological mechanisms. This approach is consistent with the use of controlled vocabularies and ontology-based annotations to structure and integrate heterogeneous toxicological knowledge [Ives et al., 2017, Mortensen et al., 2022]. The parent–child relationships were defined according to the biological scope of each reported toxicity effect and are summarized in Table **S6**. Accordingly, the hierarchy represents relationships among annotation scopes and should not be interpreted as implying a single shared toxicological mechanism among endpoints.

The root category *Toxic* represents generic adverse effects, whereas *Cytotoxic*, *Neurotoxic*, *Embryotoxic*, and *Ichthyotoxic* represent more specific biological contexts. *Hemolytic*, *Cytolysis*, and *Anti mammalian cells* were operationally assigned as subclasses of *Cytotoxic* because they describe toxicity directed toward specific cellular contexts, with hemolysis referring specifically to erythrocyte lysis and cytolysis to cell lysis or membrane-disruptive activity [Spaller et al., 2013, Wimley, 2010]. Endpoints with limited current evidence were retained when they represented distinct toxicity concepts explicitly reported by the curated sources. Their inclusion in the ontology preserves biological and terminological coverage but does not necessarily imply sufficient class representation for supervised binary benchmarking.

Supplementary Table S6: Ontology endpoint definitions used for terminology harmonization and endpoint assignment. The table summarizes the standardized toxicity endpoints used in the resource, their position in the toxicity ontology, and the operational interpretation used during annotation harmonization.

Standard endpoint	Parent class	Operational definition	Representative source terms
Toxic	Root	Generic toxicity category used for peptides reported to produce an adverse biological effect, or for sequences with positive evidence in any child toxicity endpoint. This class provides the root level for ontology-guided integration [Sharma et al., 2022].	toxic; toxicity; toxic peptide; toxin
Cytotoxic	*Toxic*	Toxicity toward cells or cell-based systems, including viability reduction, membrane damage, or cell death in eukaryotic or mammalian cellular contexts. Positive evidence from cytotoxicity-associated subclasses may be propagated to this parent category [Aslantürk, 2017].	cytotoxic; cytotoxicity
Hemolytic	*Cytotoxic*	Toxicity associated with erythrocyte damage or red blood cell lysis. This endpoint is represented as a cytotoxicity-associated subclass because hemolysis reflects cell membrane disruption in erythrocytes [Spaller et al., 2013].	hemolytic; haemolytic; hemolysis; haemolysis; hemotoxicity
Cytolysis	*Cytotoxic*	Cell lysis or membrane-disruptive activity reported as a toxicity-related effect. This category was used when the annotation specifically referred to cell lysis rather than general cytotoxicity [Wimley, 2010].	cytolytic; cytolysis; cell lysis; lytic activity; membranolytic activity

Continued on next page

Standard endpoint	Parent class	Operational definition	Representative source terms
Anti mammalian cells	*Cytotoxic*	Toxicity directed toward mammalian cells, including reduced cell viability, cellular damage, or other adverse effects explicitly reported in mammalian cellular systems. This endpoint was represented as a cytotoxicity-associated subclass because the reported effect is defined by toxicity toward mammalian cells [Matsuzaki, 2009].	anti mammalian cells; cytotoxicity in mammalian cells; toxic to mammalian cells; antimammalian cells
Neurotoxic	*Toxic*	Toxicity affecting neurons, neural cells, or the nervous system. This class was assigned only when the source annotation explicitly indicated neurotoxicity or toxin-like effects on neural systems [McQueen, 2017].	neurotoxic; neurotoxicity; neurotoxin
Embryotoxic	*Toxic*	Toxicity affecting embryos or developmental stages. This endpoint was used for annotations explicitly linked to embryo viability, developmental toxicity, or embryotoxic effects [Baker et al., 2018].	embryotoxic; embryotoxicity
Ichthyotoxic	*Toxic*	Toxicity affecting fish or fish-derived biological systems. This endpoint was used when the source annotation explicitly indicated fish toxicity, piscicidal activity, or toxicity in fish models [Bambino and Chu, 2017].	ichthyotoxic; ichthyotoxicity

S2.2 Evidence-state definitions and conflict resolution

After terminology harmonization, source-specific qualitative annotations associated with the same peptide–endpoint pair were aggregated into one mutually exclusive state in the final sequence-level pivot. The evidence model distinguishes direct endpoint evidence, unresolved or conflicting evidence, source-associated records without an explicit class assignment, and the absence of information for a specific endpoint. Table **S7** summarizes the five states used in MAOMAO. Quantitative toxicity measurements were retained separately and were not encoded using these evidence states.

Supplementary Table S7: Evidence states used in the final MAOMAO sequence-level pivot.

Code	State	Assignment and interpretation
1	*Positive*	Direct positive evidence for the endpoint, or positive evidence propagated from a child endpoint when the parent endpoint was not ambiguous.
0	*Negative*	Direct endpoint-specific negative evidence for the corresponding toxicity effect. Negative evidence was not propagated through the hierarchy.
2	*Ambiguous*	Conflicting or otherwise unresolved evidence for the same sequence–endpoint pair. Endpoint-specific ambiguity was retained and could not be overwritten by hierarchical positivity.
3	*Unlabeled*	The sequence was retained from a source associated with the endpoint, but no explicit positive or negative assignment was available for that record.
999	*No information*	No annotation was available for that endpoint. This code is distinct from both negative and unlabeled evidence.

When only positive evidence was available, the integrated state was positive; when only direct negative evidence was available, the state was negative. Peptide–endpoint pairs supported by both positive and negative annotations, or by evidence that could not be resolved without unsupported interpretation, were classified as ambiguous. Unlabeled states were assigned only when a sequence was explicitly retained by an endpoint-associated source without a class assignment. All remaining sequence–endpoint combinations in the complete cross-product were encoded as no information. Distinct quantitative toxicity measurements associated with the same peptide were preserved independently and did not contribute to qualitative ambiguity assignment.

Negative evidence was additionally characterized at the provenance level as *strong* or *weak*. Strong negatives corresponded to records for which the absence of toxicity was explicitly supported by experimental or curated evidence. Weak negatives originated from resources in which negative sequences were randomly selected or lacked explicit evidence confirming the absence of the effect. Evidence strength was retained in the supporting metadata and provenance records and was not treated as an additional pivot state.

S2.3 Hierarchy propagation rules and audit statistics

Hierarchy application followed a positive-only policy. Positive support could propagate from a more specific endpoint to its parent, whereas negative, ambiguous, unlabeled, and no-information states were never propagated. Direct ambiguity at the parent endpoint blocked hierarchy-derived positivity, thereby preserving unresolved endpoint-specific evidence. The rules applied during final annotation resolution are summarized in Table **S8**.

Supplementary Table S8: Terminology-harmonization and hierarchy-propagation rules applied during MAOMAO construction.

Rule	Source endpoint(s)	Target endpoint	Condition and action
R1	*Cytotoxic*, *Neurotoxic*, *Embryotoxic*, or *Ichthyotoxic*	*Toxic*	Positive child support set the parent to positive unless *Toxic* contained direct ambiguous evidence.
R2	*Hemolytic*, *Cytolysis*, or *Anti mammalian cells*	*Cytotoxic*	Positive child support set the parent to positive unless *Cytotoxic* contained direct ambiguous evidence.
R3	Source terms *cytolysis* and *cytolytic*	*Cytolysis*	Equivalent source terminology was harmonized before evidence aggregation and final state resolution.

The hierarchy audit records every sequence–parent-endpoint pair with positive child support and indicates whether the parent state was inferred through hierarchy propagation, was already positive before hierarchy application, or remained ambiguous because direct parent-level ambiguity blocked propagation. Table **S9** summarizes the hierarchy audit.

Supplementary Table S9: Summary of hierarchy application in MAOMAO.

Target endpoint	Positive child support	Rows inferred	Already positive	Blocked by ambiguity
Cytotoxic	7,745	6,548	355	842
Toxic	9,421	5,389	2,106	1,926
**Total**	**17,166**	**11,937**	**2,461**	**2,768**

S2.4 Final endpoint-state composition

The final sequence-level pivot contains 71,857 unique peptide sequences and eight endpoint columns, yielding 574,856 sequence–endpoint combinations. Table **S10** reports the mutually exclusive non-missing evidence-state counts after hierarchy application. All values in the table, including the *Total* row, correspond to sequence–endpoint states rather than unique peptide sequences. The *Available states* column includes positive, negative, ambiguous, and unlabeled states and therefore represents all sequence–endpoint combinations not encoded as no information (999).

Across all endpoints, 143,420 sequence–endpoint combinations contained an available evidence state. Of the 15,717 ambiguous sequence–endpoint states, 13,177 unique peptide sequences had at least one ambiguous endpoint, because a single sequence may be ambiguous for more than one endpoint. The remaining 431,436 sequence–endpoint combinations were encoded as no information (999).

Supplementary Table S10: Final non-missing evidence-state counts for each MAOMAO toxicity endpoint after hierarchy application. The *Available states* column includes positive, negative, ambiguous, and unlabeled states and excludes no-information states encoded as 999.

Endpoint	Positive	Negative	Ambiguous	Unlabeled	Available states
Toxic	13,824	35,487	7,769	0	57,080
Cytotoxic	7,122	17,238	1,094	0	25,454
Hemolytic	4,714	19,350	6,852	5,044	35,960
Cytolysis	341	2	0	0	343
Anti mammalian cells	3,664	17,729	0	0	21,393
Neurotoxic	1,481	1,700	2	0	3,183
Embryotoxic	2	0	0	0	2
Ichthyotoxic	5	0	0	0	5
**Total**	**31,153**	**91,506**	**15,717**	**5,044**	**143,420**

S2.5 Positive endpoint co-annotation analysis

Positive endpoint co-annotation was evaluated across the seven non-root toxicity endpoints using the final sequence-level pivot after hierarchy application. The root endpoint *Toxic* was excluded because it represents

the broadest toxicity category and its positive states may aggregate evidence from multiple child branches through hierarchy propagation. *Cytotoxic* was retained as an intermediate endpoint because it is also represented as an explicit toxicity endpoint in the curated sources; however, its overlaps with *Hemolytic*, *Cytolysis*, and *Anti mammalian cells* may partly reflect hierarchy-derived positive states. Only states encoded as positive were considered; negative, ambiguous, unlabeled, and no-information states were excluded.

A total of 9,421 unique peptide sequences had at least one positive annotation across the seven evaluated endpoints. Of these, 7,331 were positive for more than one endpoint, corresponding to 77.82% of sequences with at least one positive non-root annotation and 10.20% of all 71,857 unique sequences in MAOMAO. Because the analysis was performed after hierarchy application, these multi-endpoint annotations include both directly supported and hierarchy-derived positive relationships.

For each endpoint pair, the number of shared positive sequences was calculated (Table **S11**). Directional percentages were obtained by dividing each pairwise count by the total number of positive sequences assigned to the corresponding row endpoint (Table **S12**). Consequently, reciprocal percentages may differ between endpoint pairs.

Supplementary Table S11: Pairwise counts of shared positive peptide sequences across the seven non-root MAOMAO toxicity endpoints after hierarchy application. Diagonal values correspond to the total number of positive sequences assigned to each endpoint.

Row endpoint	Anti mammalian cells	Cytotoxic	Embry-otoxic	Ichthy-otoxic	Neurotoxic	Hemolytic	Cytolysis
Anti mammalian cells	3,664	3,024	0	0	7	775	117
Cytotoxic	3,024	7,122	0	3	20	4,128	247
Embryotoxic	0	0	2	0	0	0	0
Ichthyotoxic	0	3	0	5	0	2	5
Neurotoxic	7	20	0	0	1,481	8	12
Hemolytic	775	4,128	0	2	8	4,714	151
Cytolysis	117	247	0	5	12	151	341

Supplementary Table S12: Directional positive co-annotation percentages across the seven non-root MAOMAO toxicity endpoints after hierarchy application. Percentages were calculated relative to the total number of positive sequences assigned to each row endpoint and are rounded to two decimal places.

Row endpoint	Anti mammalian cells	Cytotoxic	Embry-otoxic	Ichthy-otoxic	Neurotoxic	Hemolytic	Cytolysis
Anti mammalian cells	100.00%	82.53%	0.00%	0.00%	0.19%	21.15%	3.19%
Cytotoxic	42.46%	100.00%	0.00%	0.04%	0.28%	57.96%	3.47%
Embryotoxic	0.00%	0.00%	100.00%	0.00%	0.00%	0.00%	0.00%
Ichthyotoxic	0.00%	60.00%	0.00%	100.00%	0.00%	40.00%	100.00%
Neurotoxic	0.47%	1.35%	0.00%	0.00%	100.00%	0.54%	0.81%
Hemolytic	16.44%	87.57%	0.00%	0.04%	0.17%	100.00%	3.20%
Cytolysis	34.31%	72.43%	0.00%	1.47%	3.52%	44.28%	100.00%

The largest pairwise overlap was observed between *Cytotoxic* and *Hemolytic*, with 4,128 shared positive sequences. These sequences represented 57.96% of all cytotoxic-positive sequences and 87.57% of all hemolytic-positive sequences. This overlap is consistent with the ontology structure and the positive-only hierarchy rule, through which positive hemolytic evidence can support the broader *Cytotoxic* endpoint. Accordingly, overlaps involving a parent endpoint and its subclasses should be interpreted in the context of the hierarchy rather than exclusively as independent co-occurrence of directly reported toxicity annotations.

S3 Metadata schema and provenance fields

To support transparency, reproducibility, and long-term reuse, the metadata generated during MAOMAO construction were organized into distributed and normalized records covering source-level, endpoint-level, final dataset-level, hierarchy-level, audit, quantitative toxicity-property, and sequence-profile information. Source-level metadata describe the original resources and their retrieval, licensing, annotation content, and record counts. Endpoint-level metadata document effect-specific filtering and evidence integration. Final

dataset-level metadata define the structure, stable sequence identifiers, and state encoding of the sequence-level pivot, whereas hierarchy- and audit-level records document terminology mappings, propagation rules, ambiguity handling, and hierarchy-derived changes. Quantitative toxicity measurements and sequence profiles are accompanied by dedicated metadata records that preserve their generation settings, provenance, correspondence with the Core sequence universe, and output structure. Missing metadata were preserved explicitly rather than inferred.

S3.1 Metadata schema

Source-level metadata keys were normalized to lowercase `snake_case` in the aggregated resource metadata. The metadata architecture preserves the relationships between the original sources, endpoint-specific integration outputs, final sequence–endpoint states, hierarchy rules, ambiguity-support records, quantitative toxicity measurements, sequence profiles, and distributed resource files. The principal fields and metadata records are summarized in Table **S13**.

Supplementary Table S13: Principal metadata fields and records used for MAOMAO harmonization, provenance tracking, and auditability.

**Field or metadata record**	**Description and allowed values**
**Source-level metadata**	
`source_type`	Type of original resource. Values include Database, Dataset, Publication, or Repository.
`resource_behavior`	Indicates whether the resource is static or dynamically updated.
`license`	License reported by the original provider; retained as *No information* when unavailable.
`publication_year`	Four-digit publication year.
`last_update_date`	Last update date reported by the source, when available.
`download_date`	Date on which the resource was accessed or downloaded.
`file_format`	Format of the retrieved or processed source file, such as CSV, TSV, XLSX, FASTA, JSON, or TXT.
`peptide_property`	Peptide activity or toxicity property represented by the source.
`annotation_content`	Annotation categories available in the source, including positive, negative, unlabeled, or quantitative records when applicable.
`unit_of_measurement`	Experimental unit reported by the source, when available.
`negative_dataset_origin`	Strategy used to define negative examples, such as curated negatives, random selection, or Swiss-Prot sampling.
`repository_or_server`	URL of the original repository, server, database, or downloadable resource.
`publication`	Literature reference, DOI, PMID, or publication URL associated with the source.
`number_of_raw_sequences`	Number of source records before source-specific cleaning or filtering.
`number_of_sequences_retained`	Number of records retained after source-specific processing.
`number_of_positive_sequences`	Number of retained positive records.
`number_of_negative_sequences`	Number of retained negative records.
`number_of_unlabel_sequences`	Number of retained records without an explicit positive or negative assignment.
`number_of_regression_sequences`	Number of retained quantitative or regression-oriented records, when applicable.
`number_of_erroneous_sequences`	Number of sequences removed because of invalid or erroneous content.
`number_of_modified_sequences`	Number of modified peptide sequences detected in the source.
`number_of_erroneous_modified_sequences`	Number of modified sequences excluded as erroneous.
`modified_sequences_included`	Indicates whether modified peptide sequences were retained.
**Endpoint-level metadata**	
`task`	Toxicity endpoint represented by the endpoint-integration record.
`generated_at`	ISO date and time when the endpoint metadata were generated.
`sources.n_unique_sequences`	Number of unique sequences contributed by each source to the endpoint-specific integration.
`filters.canonical_residues`	Canonical-residue filter configuration and application status.
`filters.length_filter`	Minimum and maximum accepted lengths and filter-application status.
`sequence_statistics.canonical_filter`	Sequence counts before and after canonical-residue filtering.
`sequence_statistics.length_filter`	Sequence counts before and after length filtering.
`sequence_statistics.length_distribution`	Minimum, maximum, mean, and median peptide lengths.
`statistics.total_sequences_final`	Number of final sequences retained for the endpoint before construction of the complete pivot.
`statistics.positive`	Summary of positive source evidence and source-label combinations.
`statistics.negative`	Summary of negative source evidence and source-label combinations.
`statistics.ambiguous`	Number and categories of ambiguous endpoint annotations.
`organism_statistics.targets`	Organism names or toxicity-target categories associated with endpoint annotations and their counts, when available.

Continued on next page

Supplementary Table S13: Principal metadata fields and records used for MAOMAO harmonization, provenance tracking, and auditability. Continued.

Field or metadata record	Description and allowed values
`evidence_negative_dataset_statistics.category`	Provenance-level characterization of negative evidence, including strong, weak, or unconfirmed negatives.
**Final dataset-level metadata**	
`dataset.name`	Name of the final sequence-level pivot.
`dataset.identifier_column`	Identifier column used in the sequence-level resource: `id`.
`dataset.identifier_format`	Deterministic sequence-derived identifier format: `sha256_<64 lowercase hexadecimal characters>`.
`dataset.identifier_algorithm`	SHA-256.
`dataset.identifier_input`	UTF-8 encoding of the normalized uppercase peptide sequence with whitespace removed.
`dataset.sequence_column`	Name of the peptide-sequence column.
`dataset.annotation_columns`	Eight controlled endpoint columns included in the pivot.
`dataset.n_unique_sequences`	Number of unique peptide sequences: 71,857.
`dataset.n_sequence_endpoint_rows`	Size of the complete sequence–endpoint cross-product: 574,856 combinations.
`dataset.label_encoding`	State encoding: 1 = positive, 0 = negative, 2 = ambiguous, 3 = unlabeled, and 999 = no information.
`dataset.state_priority`	States that are ambiguous for a specific endpoint cannot be overwritten by hierarchical positivity.
`dataset.negative_evidence_policy`	Negative evidence is endpoint-specific and is not propagated through the hierarchy.
`dataset.missing_information_policy`	Code 999 denotes unavailable endpoint information and is distinct from negative and unlabeled evidence.
**Hierarchy- and audit-level metadata**	
`controlled_vocabulary_and_hierarchy.version`	Version of the MAOMAO controlled vocabulary and hierarchy.
`hierarchy_policy.positive_propagation`	Indicates that positive evidence can propagate from a child endpoint to its parent.
`hierarchy_policy.negative_propagation`	Indicates that negative evidence is not propagated.
`hierarchy_policy.ambiguity_overrides_hierarchy`	Indicates that direct parent ambiguity blocks hierarchy-derived positivity.
`terms.term`	Harmonized or source-specific toxicity term.
`terms.label_type`	Role of the term, such as broad, intermediate, specific, or source-specific.
`terms.parent_terms`	Parent endpoint or endpoints associated with a controlled term.
`terms.harmonized_to`	Controlled endpoint to which a source-specific synonym was mapped.
`rules_applied.rule_id`	Identifier of the applied terminology or hierarchy rule.
`rules_applied.source_terms`	Child or source terms that trigger the rule.
`rules_applied.target_term`	Parent or harmonized endpoint modified by the rule.
`rules_applied.condition`	Logical condition under which the rule is applied.
`rules_applied.action`	State change or terminology mapping performed by the rule.
`rules_applied.blocked_when`	Endpoint-specific state that prevents propagation, when applicable.
`rules_applied.propagation_policy`	Propagation policy; the hierarchy rules use positive-only propagation.
`ambiguity.support_file`	File containing endpoint-specific support summaries for ambiguous sequence–endpoint states.
`output_files`	File-level inventory containing descriptions, dimensions, checksums, and column information for principal outputs.
**Quantitative toxicity-property metadata**	
`identifier_algorithm`	Sequence-matching identifier algorithm used for quantitative records: SHA-256.
`identifier_input`	Normalized peptide sequence representation used to generate the sequence identifier.
`sequence_policy`	Canonical-residue and sequence-length requirements used for cross-referencing quantitative records with the Core sequence universe.
`unit_policy`	Reported measurement units are preserved and no automatic unit conversion is performed.
`compact_property_columns`	Quantitative toxicity-property columns included in the compact sequence-level representation: `hc50`, `lc50`, `ld50`, and `mhc`.
`compact_missing_value_code`	Missing quantitative property code used in the compact representation.
`measurement_records`	Number of parsed quantitative toxicity measurement records.
`measurement_unique_sequences`	Number of unique peptide sequences represented by quantitative measurements.
`matched_records`	Number of quantitative records matched to the Core sequence universe.
`matched_unique_sequences`	Number of unique quantitative-measurement sequences matched to the Core sequence universe.
`unmatched_records`	Number of quantitative records that could not be matched to the Core sequence universe.
`unmatched_unique_sequences`	Number of unique quantitative-measurement sequences absent from the Core sequence universe.

Continued on next page

Supplementary Table S13: Principal metadata fields and records used for MAOMAO harmonization, provenance tracking, and auditability. Continued.

Field or metadata record	Description and allowed values
`outputs`	File names for the long-form measurement table, compact sequence-level characterization, cross-reference audit, unmatched-sequence audit, and metadata record.
**Sequence-profile metadata and schema**	
`sequence_profiles/metadata.json`	Metadata describing sequence-profile provenance, file paths, coverage, selected descriptor fields, and checksums.
`sequence_profiles/sequence_card_schema.json`	JSON Schema defining the structure and allowed fields of an individual sequence card.
`sequence_profiles/sequence_cards.jsonl.gz`	Machine-readable collection containing one compact JSON object per normalized MAOMAO sequence.
`sequence_profiles/sequence_activity_evidence.csv.gz`	Detailed sequence–endpoint evidence table supporting the compact sequence profiles.
`sequence_profiles/sequence_source_evidence.csv.gz`	Detailed sequence–endpoint–source evidence table preserving source-level provenance for sequence profiles.

S3.2 Source metadata coverage

Metadata availability was evaluated across the 56 sources integrated into MAOMAO. As summarized in Table **S14**, publication references, repository or server information, download dates, file formats, source types, and resource behavior were available for all sources. Annotation content and last-update dates were available for 55 sources (98.21%). In contrast, explicit licensing information was available for 15 sources (26.79%), and source-level experimental units of measurement were documented for only 3 sources (5.36%). Missing values were retained as unavailable information rather than being inferred from related publications or external resources.

Supplementary Table S14: Metadata-field availability across the 56 sources integrated into MAOMAO.

Metadata field	Available	Total	Coverage
Publication	56	56	100%
Repository or server	56	56	100%
Download date	56	56	100%
File format	56	56	100%
Source type	56	56	100%
Resource behavior	56	56	100%
Annotation content	55	56	98.21%
Last-update date	55	56	98.21%
License	15	56	26.79%
Unit of measurement	3	56	5.36%

The metadata-coverage results reflect the information explicitly reported by the original resources and available at the time of retrieval. Consequently, low coverage for fields such as licensing and source-level measurement units represents limitations of the source documentation rather than values omitted during MAOMAO harmonization.

S3.3 Source access and licensing audit

Access and licensing information was consolidated for the 56 resources integrated into MAOMAO (Table **S15**). An explicit license was recorded for 15 resources (26.79%), whereas no explicit license was identified in the recorded metadata for the remaining 41 resources. The table summarizes the recorded retrieval method, last-update information, reported license or terms, and a provisional assessment of redistribution and attribution requirements. Interpretations of the MIT License, Apache License 2.0, Creative Commons Attribution 4.0 International License, GNU General Public License, and Database Contents License/Open Database License conditions were based on their respective official license texts [Open Source Initiative, 2026, Apache Software Foundation, 2004, Creative Commons, 2013, Free Software Foundation, 2007, Open Data Commons, 2008, 2009]. Because a license displayed at the repository level may apply to software or documentation rather than to the associated data files, all redistribution assessments should be interpreted as provisional and subject to source-level verification.

Supplementary Table S15: Source retrieval and provisional licensing audit for the 56 resources integrated into MAOMAO. Reported licenses reproduce the information recorded in the source metadata.

Source	Last update	Retrieval method	Reported license or terms	Redistribution status	Attribution or conditions
AMPDB v1	2023-05-03–2023-05-26	Database/web-server download	No information	Not established	Not established
AMPDeep	2022-09-28	Repository download	MIT	Permitted, subject to license terms	Copyright and permission notice
Abdelbaky et al.	2024-06-29	Repository download	No information	Not established	Not established
Almotairi et al.	2024-06-28	Repository download	No information	Not established	Not established
BIOPEP-UWM	2025-08-27	Database/web-server download	No information	Not established	Not established
Bhatnagar et al.	2024-10-30	Supplementary-file retrieval	No information	Not established	Not established
BiToxNet	2025-11-09	Repository download	No information	Not established	Not established
CAPTP	2025-09-24	Repository download	No information	Not established	Not established
CICERON	2024-04-30	Repository download	No information	Not established	Not established
ConsAMPHemo	2024-09-28	Repository download	No information	Not established	Not established
DRAMP	2025-07-16	Database/web-server download	Creative Commons Attribution 4.0	Permitted with attribution	Credit, license reference, and change indication
HAPPENN	No information	Database/web-server download	No information	Not established	Not established
HEPAD	2024-04-21	Repository download	No information	Not established	Not established
HLPpred-Fuse	2020-04-20	Database/web-server download	No information	Not established	Not established
HMAMP-main	2025-09-03	Repository download	No information	Not established	Not established
HemoDL	2023-04-18	Repository download	No information	Not established	Not established
HemoFuse	2024-09-19	Repository download	No information	Not established	Not established
HemoPI	2016-03-08	Database/web-server download	No information	Not established	Not established
HemoPI2.0	2025-02-05	Database/web-server download	No information	Not established	Not established
Hemolytic-Pred	2023-07-05	Supplementary-file retrieval	No information	Not established	Not established
Hemolytik	2014-01-01	Database/web-server download	No information	Not established	Not established
HyPepTox-Fuse	2025-07-24	Repository download	Apache 2.0	Permitted, subject to license terms	License and applicable NOTICE retention
Karasev et al.	2024-12-01	Supplementary-file retrieval	No information	Not established	Not established
MultiPep	2023-03-30	Repository download	MIT	Permitted, subject to license terms	Copyright and permission notice
Multitox	2025-05-19	Repository download	No information	Not established	Not established
NTXpred	2007-11-01	Database/web-server download	No information	Not established	Not established
NTXpred2	2025-02-21	Database/web-server download	GNU General Public License	Permitted under GPL conditions	License notices and applicable copyleft/source obligations
PLPTP	2025-02-23	Repository download	No information	Not established	Not established
Pep-Lab__db	2023-01-10	Database/web-server download	No information	Not established	Not established
PeptiTox	2025-03-28	Repository download	No information	Not established	Not established
Peptipedia2.0	2024-03-23	Database/web-server download	DbCL	Conditional under DbCL/ODbL terms	Applicable attribution and database-license conditions
Plisson et al.	2020-08-24	Repository download	MIT	Permitted, subject to license terms	Copyright and permission notice
ProToxin	2025-06-25	Database/web-server download	No information	Not established	Not established
QSVM-PEPTIDE	2024-09-19	Repository download	No information	Not established	Not established
SATPdb	2015-11-01	Database/web-server download	No information	Not established	Not established
StrucToxNet	2025-06-05	Repository download	No information	Not established	Not established
TPpred-LE	2023-01-31	Database/web-server download	No information	Not established	Not established
ToxDL 2.0	2025-04-02	Database/web-server download	No information	Not established	Not established
ToxGIN	2025-01-18	Repository download	No information	Not established	Not established
ToxIBTL	2022-01-24	Repository download	No information	Not established	Not established
ToxMSRC	2025-08-25	Repository download	No information	Not established	Not established
ToxTeller	2023-12-25	Repository download	No information	Not established	Not established
ToxiPep	2025-05-20	Repository download	No information	Not established	Not established

Continued on next page

Supplementary Table S15 – *Continued from previous page*

Source	Last update	Retrieval method	Reported license or terms	Redistribution status	Attribution or conditions
ToxinPred	2013-09-13	Database/web-server download	No information	Not established	Not established
ToxinPred 2.0	2022-05-21	Database/web-server download	GNU General Public License	Permitted under GPL conditions	License notices and applicable copyleft/source obligations
ToxinPred 3.0	2024-07-21	Database/web-server download	GNU General Public License	Permitted under GPL conditions	License notices and applicable copyleft/source obligations
UniDL4BioPep	2024-11-22	Repository download	MIT	Permitted, subject to license terms	Copyright and permission notice
Zhao et al.	2021-05-26	Supplementary-file retrieval	Creative Commons Attribution 4.0	Permitted with attribution	Credit, license reference, and change indication
csm-toxin	2023-01-30	Repository download	No information	Not established	Not established
hemolytik 2.0	2014-05-14	Database/web-server download	No information	Not established	Not established
hemonet	2021-03-11	Repository download	No information	Not established	Not established
iAMPCN	2023-03-02	Repository download	No information	Not established	Not established
peptidereactor	2021-05-25	Repository download	MIT	Permitted, subject to license terms	Copyright and permission notice
tAMPer	2023-11-19	Repository download	GNU General Public License	Permitted under GPL conditions	License notices and applicable copyleft/source obligations
Hemolytik2.0_2026	2026-05-14	Repository download	GNU General Public License	Permitted under GPL conditions	License notices and applicable copyleft/source obligations
PepNet	2024-08-06	Repository download	Creative Commons Attribution 4.0	Permitted with attribution	Credit, license reference, and change indication

Note. "No information" indicates that an explicit license was not recorded in the source metadata and must not be interpreted as public-domain status or permission to redistribute. The redistribution and attribution columns constitute a provisional assessment based on the reported license label and require source-level verification that the stated license applies to the downloaded data rather than only to repository software or documentation. Recorded retrieval methods correspond to repository downloads, database or web-server downloads, and supplementary-file retrieval. Associated publications are provided in the reference list, whereas source locations are preserved in the released provenance metadata.

S4 Computational resource generation and file organization

S4.1 Descriptor Layer generation

A single descriptor table was generated from the final 71,857-sequence MAOMAO pivot to provide reusable physicochemical and sequence-derived features across all toxicity endpoints. Descriptor extraction was performed with the Roxy framework v0.1.0 [KrenAI-Lab, 2026b] using the peptide sequence column as input. No single target column was specified because the resource contains eight endpoint-state columns. AAindex features were not enabled; the descriptor configuration retained the sequence-global feature set.

The output table contains the sequence identifier, peptide sequence, eight endpoint-state columns, and 41 descriptor columns, resulting in 71,857 rows and 51 columns. The descriptor matrix is distributed as `descriptor_layer/dataset_characterization/sequence_descriptors.csv`, together with `descriptor_layer/dataset_characterization/metadata.json`, which documents the generation settings, state encoding, output dimensions, descriptor names, and quality-control results.

Supplementary Table S16: Descriptor Layer generation settings.

Parameter	Value
Descriptor framework	Roxy v0.1.0
Input resource	Final MAOMAO sequence-level pivot
Input rows	71,857 unique peptide sequences
Identifier column	`id`
Sequence column	`sequence`
Endpoint-state columns	`toxic`, `cytotoxic`, `hemolytic`, `cytolysis`, `anti_mammalian_cells`, `neurotoxic`, `embryotoxic`, and `ichthyotoxic`
Feature set	Sequence-global descriptors
AAindex features	Disabled

Continued on next page

Supplementary Table S16 – *Continued from previous page*

Parameter	Value
Target column	None; all eight endpoint-state columns were retained
Descriptor columns	41
Output dimensions	71,857 rows × 51 columns
Descriptor output	`descriptor_layer/dataset_characterization/sequence_descriptors.csv`
Metadata output	`descriptor_layer/dataset_characterization/metadata.json`

The 41 descriptors are organized into the groups summarized in Table **S17**.

Supplementary Table S17: Sequence-derived descriptors included in the MAOMAO Descriptor Layer.

Descriptor group	Descriptor columns	Interpretation
Sequence length	`length`	Number of amino-acid residues in the peptide.
Amino-acid composition	`aa_frac_A`, `aa_frac_C`, `aa_frac_D`, `aa_frac_E`, `aa_frac_F`, `aa_frac_G`, `aa_frac_H`, `aa_frac_I`, `aa_frac_K`, `aa_frac_L`, `aa_frac_M`, `aa_frac_N`, `aa_frac_P`, `aa_frac_Q`, `aa_frac_R`, `aa_frac_S`, `aa_frac_T`, `aa_frac_V`, `aa_frac_W`, `aa_frac_Y`	Fraction of each canonical amino acid in the sequence.
Residue-class composition	`frac_aromatic`, `frac_positive`, `frac_negative`, `frac_polar`, `frac_nonpolar`	Fractions of residues grouped by physicochemical class.
Hydrophobicity and hydropathy	`gravy_kd`, `hydropathy_eisenberg`	Mean sequence hydropathy according to the Kyte–Doolittle and Eisenberg scales.
Structural propensity and disorder	`top_idp_mean`, `helix_propensity_mean`, `sheet_propensity_mean`	Mean disorder, helix, and sheet propensity values.
Interaction propensity	`boman_index`	Estimated protein-binding or interaction potential.
Charge-related properties	`net_charge_pH`, `fcr`, `ncpr`	Estimated net charge, fraction of charged residues, and net charge per residue.
Hydrogen-bonding capacity	`donors_per_residue`, `acceptors_per_residue`	Estimated hydrogen-bond donors and acceptors normalized by sequence length.
Sequence complexity	`aa_entropy`, `lc_k1`, `lc_k2`, `lc_k3`	Amino-acid entropy and low-complexity measures at increasing word lengths.

S4.2 Numerical representation extraction

Protein language model representations were generated for the complete harmonized MAOMAO sequence collection and were subsequently reused across endpoint-specific benchmark construction. Embeddings were obtained with the Sylphy v0.2.0 framework [KrenAI-Lab, 2026c] using pretrained protein language models without task-specific fine-tuning. Residue-level outputs from the final embedding layer were mean-pooled to obtain one fixed-length vector for each of the 71,857 peptide sequences.

Embedding extraction was performed using `cuda` with `fp32` precision and a batch size of 16. For each protein language model, `embeddings.csv` stores the resulting sequence-level numerical matrix, whereas `full_data.csv` preserves the correspondence between the stable sequence identifier (`id`), peptide sequence, endpoint states, and embedding rows. The numerical-representation root is `numerical_representation_data/maomao/`, with protein language model embeddings stored under the `sylphy_embedding/` branch. The extraction settings are summarized in Table **S18**.

Supplementary Table S18: Embedding extraction settings used for the protein language model representations.

Parameter	Value
Framework	Sylphy v0.2.0
Input resource	Complete harmonized MAOMAO sequence collection
Input sequences	71,857 unique peptide sequences
Identifier column	`id`
Model state	Pretrained, without task-specific fine-tuning
Representation layer	Final embedding layer
Pooling strategy	Mean pooling across residue-level embeddings
Device	`cuda`

Continued on next page

Supplementary Table S18 – *Continued from previous page*

Parameter	Value
Precision	`fp32`
Batch size	16
Output format	CSV
Representation root	`numerical_representation_data/maomao/sylphy_embedding/`
Main output file	`embeddings.csv`
Companion file	`full_data.csv`

S4.3 Supported protein language model embeddings

The MAOMAO Embedding Layer provides ten precomputed sequence-level embedding matrices derived from pretrained protein language models. These representations cover five model families—Ankh, ESM2, ESM-C, Mistral-Prot, and ProtTrans—and include architectures with different parameter scales and output dimensions. Each matrix assigns a fixed-length numerical representation to the 71,857 peptide sequences in the final MAOMAO pivot, allowing the same curated sequence collection to be examined across alternative learned representation spaces without requiring users to regenerate the embeddings.

Table **S19** summarizes the model variant, protein language model family, relative file location, and embedding dimension of each distributed matrix. The available representations range from 320 to 1,536 features per peptide sequence. The four ESM2 variants provide representations obtained from models of increasing scale, whereas the Ankh, ESM-C, Mistral-Prot, and ProtTrans models broaden the representation layer across distinct pretrained protein language model families.

Supplementary Table S19: Protein language model embeddings included in the MAOMAO Embedding Layer. File paths are relative to `numerical_representation_data/maomao/sylphy_embedding/`.

Model	Family	Embedding file	Dimension
Ankh2-ext1 [Elnaggar et al., 2023]	Ankh	`ankh2_ext1/embeddings.csv`	1536
Ankh3-large [Alsamkary et al., 2025]	Ankh	`ankh3_large/embeddings.csv`	1536
ESM2 t6 8M UR50D [Lin et al., 2022]	ESM2	`esm2_t6_8M_UR50D/embeddings.csv`	320
ESM2 t12 35M UR50D [Lin et al., 2022]	ESM2	`esm2_t12_35M_UR50D/embeddings.csv`	480
ESM2 t30 150M UR50D [Lin et al., 2022]	ESM2	`esm2_t30_150M_UR50D/embeddings.csv`	640
ESM2 t33 650M UR50D [Lin et al., 2022]	ESM2	`esm2_t33_650M_UR50D/embeddings.csv`	1280
ESM-C 300M [Candido et al., 2026]	ESM-C	`esmc_300m/embeddings.csv`	960
Mistral-Prot v1 134M [Mourad, 2024]	Mistral-Prot	`mistral_prot_v1_134M/embeddings.csv`	768
ProtBERT [Elnaggar et al., 2021]	ProtTrans	`prot_bert/embeddings.csv`	1024
ProtT5-XL-UniRef50 [Elnaggar et al., 2021]	ProtTrans	`prot_t5_xl_uniref50/embeddings.csv`	1024

S4.4 One-hot baseline representation

A conventional one-hot representation was generated using Sylphy v0.2.0 as a baseline and was distributed separately from the protein language model embeddings. In this encoding, each amino acid at a given sequence position is represented by a binary indicator vector over the amino-acid alphabet, providing a direct sequence-based representation without learned contextual information [Jing et al., 2019]. The encoded matrix contains representations for the same 71,857 peptide sequences included in the final MAOMAO pivot and is stored under `numerical_representation_data/maomao/sylphy_one_hot/one_hot/encoded.csv`, whereas `full_data.csv` retains the corresponding sequence identifiers, peptide sequences, endpoint states, and row correspondence.

Together, the ten protein language model embedding matrices and the one-hot baseline define the eleven numerical representations available for reconstructing representation-specific benchmark datasets. All representations cover the complete 71,857-sequence MAOMAO collection and can be linked to the corresponding Core and Benchmark Layer records through the sequence identifier.

S4.5 Benchmark partition generation

Benchmark-ready partitions were generated with BioSieve v0.1.0 [KrenAI-Lab, 2026a] for toxicity endpoints containing sufficient positive and negative sequence counts to support binary five-fold partitioning. Eligibility was assessed using the final endpoint states after hierarchy application. Only sequences encoded as positive or negative for the corresponding endpoint were included in the binary benchmark datasets; ambiguous,

unlabeled, and no-information states were excluded. As summarized in Table **S20**, five endpoints met the predefined eligibility criteria: *Toxic*, *Cytotoxic*, *Hemolytic*, *Neurotoxic*, and *Anti mammalian cells*. *Cytolysis*, *Embryotoxic*, and *Ichthyotoxic* were not included because their final positive–negative distributions were insufficient for the predefined five-fold design.

Supplementary Table S20: Endpoint eligibility for binary benchmark partitioning. Positive and negative counts correspond to the final sequence-level pivot after hierarchy application. Ambiguous, unlabeled, and no-information states were excluded from the binary benchmark datasets.

Endpoint	Positive	Negative	Eligible	Benchmark interpretation
Toxic	13,824	35,487	Yes	Sufficient positive and negative examples for the predefined design.
Cytotoxic	7,122	17,238	Yes	Sufficient positive and negative examples for the predefined design.
Hemolytic	4,714	19,350	Yes	Sufficient positive and negative examples for the predefined design.
Neurotoxic	1,481	1,700	Yes	Sufficient positive and negative examples for the predefined design.
Anti mammalian cells	3,664	17,729	Yes	Sufficient positive and negative examples for the predefined design.
Cytolysis	341	2	No	The negative class is insufficient for the predefined five-fold design.
Embryotoxic	2	0	No	No negative endpoint-specific examples are available.
Ichthyotoxic	5	0	No	No negative endpoint-specific examples are available.

For each eligible endpoint, random and stratified partitions were generated independently for each of 30 random seeds. Because these partitioning strategies operate on sequence identifiers and endpoint labels rather than on numerical features, the resulting train, validation, and test assignments were generated independently of the 11 numerical representations. Random partitioning did not explicitly preserve endpoint-label proportions, whereas stratified partitioning preserved class proportions across the generated subsets. The same set of random seeds was used across endpoints and partitioning strategies to support reproducible and comparable partition generation.

Each partition configuration was organized as a five-fold design. For every fold, the corresponding `train.csv`, `val.csv`, and `test.csv` files store the sequence identifier and binary endpoint label. Because all numerical representations use the same stable sequence identifiers, representation-specific benchmark datasets can be reconstructed by joining the partition files with any of the ten protein language model embeddings or the one-hot baseline using `id` as the key. The complete partition-generation settings are summarized in Table **S21**.

Supplementary Table S21: Benchmark partition generation settings used in MAOMAO.

Parameter	Value
Partitioning framework	BioSieve v0.1.0
Eligible endpoints	*Toxic*, *Cytotoxic*, *Hemolytic*, *Neurotoxic*, and *Anti mammalian cells*
Endpoint classes	Positive and negative
Excluded evidence states	Ambiguous, unlabeled, and no information
Compatible numerical representations	Ten protein language model embeddings and one one-hot baseline
Representation count	11
Split source	Non-reduced endpoint-specific binary datasets: `no_reduced`
Enabled strategies	`random_kfold` and `stratified_kfold`
Number of folds	5
Random seeds	30
Seed file	`general_configs/random_seeds_30.csv`
Shuffle	Enabled
Validation size	0.1
Stratification variable	Endpoint label for `stratified_kfold`
Required fold files	`train.csv`, `val.csv`, and `test.csv`
Stored fold columns	`id` and `label`
Validation criteria	At least two classes overall and at least two classes in every generated split

Continued on next page

Supplementary Table S21 – *Continued from previous page*

**Parameter**	**Value**
Invalid split handling	Invalid configurations are documented without stopping the complete workflow
Stored partition configuration count	300
Stored fold count	1,500
Reconstructable representation-specific configuration count	3,300
Reconstructable representation-specific fold count	16,500

The number of physically stored partition configurations is determined by the number of eligible endpoints, random seeds, and partitioning strategies:

$$N_{\text{stored}} = 5 \text{ endpoints} \times 30 \text{ seeds} \times 2 \text{ strategies} = 300. \tag{S1}$$

Because each stored configuration contains five folds, the number of stored fold instances is:

$$N_{\text{stored folds}} = 300 \text{ configurations} \times 5 \text{ folds} = 1{,}500. \tag{S2}$$

The stored partitions are independent of the numerical representation and can therefore be combined with any of the 11 numerical representations distributed in MAOMAO. The number of reconstructable representation-specific benchmark configurations is:

$$N_{\text{reconstructed}} = 300 \text{ configurations} \times 11 \text{ representations} = 3{,}300. \tag{S3}$$

The corresponding number of reconstructable representation-specific train–validation–test fold instances is:

$$N_{\text{reconstructed folds}} = 1{,}500 \text{ folds} \times 11 \text{ representations} = 16{,}500. \tag{S4}$$

S4.6 File organization

The computational artifacts described in this section are distributed across three dedicated MAOMAO resource layers: the Descriptor Layer, the Embedding Layer, and the Benchmark Layer. These distribution layers separate sequence characterization, numerical sequence representations, and benchmark-ready data partitions while preserving stable sequence identifiers that allow records to be linked across resource components. The Core and Documentation Layers contain the harmonized biological resource, provenance records, sequence profiles, and supporting documentation and are described separately.

Descriptor resources are distributed under `descriptor_layer/`. The `dataset_characterization/` directory contains the global descriptor matrix and its accompanying metadata. The `sequence_descriptors.csv` file contains sequence identifiers, peptide sequences, the eight endpoint-state columns, and the 41 physicochemical and sequence-derived descriptors. The accompanying `metadata.json` documents descriptor-generation settings, output dimensions, descriptor definitions, state encoding, and quality-control information.

```
descriptor_layer/
  README.md
  dataset_characterization/
    metadata.json
    sequence_descriptors.csv
```

Precomputed numerical representations are distributed under `embedding_layer/`. Protein language model embeddings and the one-hot baseline are organized in separate branches under `numerical_representation_data/maomao/`. For each protein language model, `embeddings.csv` contains the fixed-length sequence-level numerical matrix, whereas `full_data.csv` preserves the correspondence between stable sequence identifiers, peptide sequences, endpoint states, and representation rows. The one-hot branch follows the same organization: `encoded.csv` contains the numerical encoding and `full_data.csv` preserves the corresponding sequence-level information.

```
embedding_layer/
  README.md
  numerical_representation_data/
    maomao/
      sylphy_embedding/
        <model_name>/
          embeddings.csv
          full_data.csv
      sylphy_one_hot/
        one_hot/
          encoded.csv
          full_data.csv
```

Benchmark-ready partitions are distributed under `benchmark_layer/split_process/`. For the random and stratified strategies included in MAOMAO, the directory hierarchy identifies the toxicity endpoint, the non-reduced binary split source, the partitioning strategy, the random seed, and the fold. Because the released partition assignments are generated independently of numerical features, no representation-specific directory is required within the Benchmark Layer. The same stored partition can therefore be combined with any of the eleven compatible numerical representations.

At the seed level, `params_split.yaml` records the partition-generation parameters, whereas `kfold_report.json` and `split_summary.csv` summarize the resulting five-fold partition. Standard-output and standard-error logs preserve execution information, and `DONE.txt` records successful workflow completion. Each fold directory contains `train.csv`, `val.csv`, and `test.csv`; these files contain only the stable sequence identifier (`id`) and the corresponding binary endpoint label (`label`).

```
benchmark_layer/
  README.md
  split_process/
    maomao_<endpoint>/
      no_reduced/
        <random_kfold|stratified_kfold>/
          seed_<seed>/
            biosieve_split.stderr.log
            biosieve_split.stdout.log
            DONE.txt
            kfold_report.json
            params_split.yaml
            split_summary.csv
            fold_00/
              train.csv
              val.csv
              test.csv
            ...
            fold_04/
              train.csv
              val.csv
              test.csv
```

Representation-specific benchmark datasets can be reconstructed by joining a selected fold-level partition with the corresponding numerical representation using `id` as the common key. For protein language model embeddings, the representation-specific information is obtained from the corresponding `full_data.csv` file under `embedding_layer/numerical_representation_data/maomao/sylphy_embedding/`; the analogous one-hot companion file is stored under the `sylphy_one_hot/one_hot/` branch.

Names enclosed in angle brackets indicate variable directory components. Thus, `<endpoint>` identifies one of the eligible binary toxicity endpoints, `<random_kfold|stratified_kfold>` identifies the partitioning strategy, `<seed>` identifies the random seed used to generate the corresponding partition, and `<model_name>` identifies the protein language model representation. The `no_reduced` directory indicates that the partitions were generated from the non-reduced endpoint-specific binary datasets.

S5 Resource contents and file summaries

This section provides a structured overview of the principal files distributed across the Core, Descriptor, Embedding, Benchmark, and Documentation Layers of MAOMAO. Each layer groups files according to their role within the overall data and reuse workflow. The Core Layer contains the harmonized sequence-level resource, endpoint assignments, provenance and audit information, quantitative toxicity measurements, and sequence-level profiles. The Descriptor Layer provides physicochemical and sequence-derived features, whereas the Embedding Layer contains numerical representations generated using protein language models and a sequence-encoding baseline. The Benchmark Layer provides fixed, representation-independent train–validation–test partitions and their associated configuration and execution records. Finally, the Documentation Layer supplies schemas, controlled-vocabulary descriptions, licensing and versioning information, release inventories, integrity records, and outputs associated with the practical usage examples. Detailed generation procedures, software settings, and processing parameters are described separately in Supplementary Section S4.

S5.1 Core Layer outputs

The Core Layer contains the final sequence-level pivot together with the files required to inspect endpoint-state composition, ambiguity support, hierarchy application, and quantitative toxicity measurements. It also includes automatically generated sequence-level profiles that integrate the principal evidence associated with each peptide. Table **S22** summarizes the principal tabular and metadata outputs.

Supplementary Table S22: Principal Core Layer and audit files.

File	Rows	Columns	Description
maomao_sequence_pivot.csv	71,857	10	Stable sequence identifiers, peptide sequences, and eight mutually exclusive endpoint-state columns.
maomao_sequence_pivot_with_toxicity_properties.csv	71,857	14	Final sequence-level pivot combined with compact hc50, lc50, ld50, and mhc fields.
maomao_toxicity_measurements.csv	169	15	Long-form quantitative toxicity measurements preserving measurement type, relation, value, error, unit, sequence information, and source provenance.
maomao_ambiguous_support.csv	13,177	7	Unique peptide sequences with at least one ambiguous endpoint and their endpoint-specific support summaries.
audit_endpoint_counts.csv	8	5	Positive, negative, ambiguous, and unlabeled counts for each controlled toxicity endpoint.
audit_hierarchy_changes.csv	17,166	10	Sequence-level audit of positive child support, hierarchy-derived assignments, ambiguity blocking, and hierarchical conflicts.
audit_toxicity_property_cross_reference.csv	5	9	Measurement-type and overall summaries of quantitative-record correspondence with the Core sequence universe.
audit_toxicity_property_unmatched_sequences.csv	6	15	Quantitative toxicity records whose peptide sequences were not retained in the final Core sequence universe.
metadata.json	—	—	Aggregated resource, source, endpoint, hierarchy, ambiguity, sequence, and file metadata.
metadata_toxicity_properties.json	—	—	Quantitative-property parsing, sequence-matching, unit, coverage, and output metadata.

The principal tabular Core outputs are distributed under:

`core_layer/processed_data/processed_data/`

The Core Layer also provides a sequence-profile collection under `core_layer/sequence_profiles/`. The primary file, `sequence_cards.jsonl.gz`, contains one compact JSON object per normalized MAOMAO peptide sequence. Each card summarizes the stable sequence identifier, peptide sequence, final endpoint states, direct-source evidence, hierarchy-derived support, negative evidence, toxicity targets when available, quantitative toxicity measurements, source provenance, and a compact subset of physicochemical descriptors.

Supporting sequence-profile resources include:

```
core_layer/sequence_profiles/
  sequence_cards.jsonl.gz
  sequence_activity_evidence.csv.gz
  sequence_source_evidence.csv.gz
  sequence_card_schema.json
  metadata.json
  CHECKSUMS.sha256
  examples/
  selected_cards/
```

The compact descriptor subset included in the sequence cards consists of `net_charge_pH`, `boman_index`, `fcr`, and `aa_entropy`. These values provide a lightweight sequence summary and do not replace the complete 41-descriptor matrix distributed in the Descriptor Layer.

S5.2 Descriptor Layer outputs

The Descriptor Layer contains one 71,857-row table combining the stable sequence identifiers, peptide sequences, eight endpoint-state columns, and 41 numerical descriptors. The accompanying metadata file records descriptor definitions, generation parameters, output dimensions, label encoding, and quality-control results.

Supplementary Table S23: Descriptor Layer file summary.

Resource component	File	Description
Descriptor matrix	`descriptor_layer/dataset_characterization/sequence_descriptors.csv`	71,857 rows and 51 columns, including 41 sequence-derived descriptors.
Descriptor metadata	`descriptor_layer/dataset_characterization/metadata.json`	Generation settings, descriptor names, output dimensions, state distributions, and quality-control information.

S5.3 Embedding Layer outputs

The Embedding Layer contains ten protein language model-derived matrices and one conventional one-hot baseline representation. Protein language model files are stored under model-specific directories, and each numerical matrix is accompanied by a file preserving identifier, sequence, endpoint-state, and row correspondence. The same stable sequence identifiers used throughout MAOMAO enable the numerical representations to be linked with Core and Benchmark Layer records.

Supplementary Table S24: Numerical representation file summary.

Resource component	Directory or file pattern	Summary
Protein language model embeddings	`embedding_layer/numerical_representation_data/maomao/sylphy_embedding/<model>/`	Ten model-specific sequence-level embedding matrices.
Main embedding file	`embeddings.csv`	One fixed-length numerical vector per peptide sequence.
Embedding companion file	`full_data.csv`	Identifier, peptide sequence, endpoint-state information, and row correspondence.
One-hot baseline	`embedding_layer/numerical_representation_data/maomao/sylphy_one_hot/one_hot/encoded.csv`	Conventional sequence encoding distributed as the eleventh numerical representation.
One-hot companion file	`embedding_layer/numerical_representation_data/maomao/sylphy_one_hot/one_hot/full_data.csv`	Identifier, peptide sequence, endpoint-state information, and row correspondence for the one-hot representation.

Together, the ten protein language model embeddings and the one-hot baseline define eleven numerical representations available for downstream reuse and reconstruction of representation-specific benchmark datasets.

S5.4 Benchmark partition resources

The Benchmark Layer contains 300 stored endpoint–seed–strategy partition configurations and 1,500 stored train–validation–test fold instances. Each stored configuration contains fifteen fold-level CSV files—three

files for each of five folds—together with six seed-level configuration, report, completion, and execution-log files. Consequently, the stored partition schema contains 6,300 files under split_process/. The fold-level files contain only stable sequence identifiers and binary endpoint labels.

By combining the stored partitions with the 11 numerical representations distributed in the Embedding Layer, users can reconstruct 3,300 representation-specific benchmark configurations and 16,500 representation-specific fold instances.

Supplementary Table S25: Benchmark partition resource summary.

**Benchmark component**	**Summary**
Benchmark directory	benchmark_layer/split_process/maomao_<endpoint>/no_reduced/
Eligible toxicity endpoints	Five: *Toxic*, *Cytotoxic*, *Hemolytic*, *Neurotoxic*, and *Anti mammalian cells*
Compatible numerical representations	Eleven: ten protein language model embeddings and one one-hot baseline
Random seeds	30
Partitioning strategies	random_kfold and stratified_kfold
Stored partition configuration count	$5 \times 30 \times 2 = 300$
Fold configuration	Five train–validation–test folds per stored configuration
Stored fold count	$300 \times 5 = 1,500$
Reconstructable representation-specific configuration count	$300 \times 11 = 3,300$
Reconstructable representation-specific fold count	$1,500 \times 11 = 16,500$
Fold-level files	train.csv, val.csv, and test.csv
Fold-level columns	id and label
Seed-level files	params_split.yaml, kfold_report.json, split_summary.csv, DONE.txt, and standard-output and standard-error logs
Files per stored configuration	21: fifteen fold-level CSV files and six seed-level files
Stored partition-schema file count	6,300 files under split_process/

S5.5 Release-level organization

MAOMAO is distributed in complementary resource layers according to the function of each component within the data-construction and reuse workflow. This modular organization separates the harmonized biological data from derived sequence descriptors, numerical representations, benchmark partitions, and supporting documentation while preserving stable sequence identifiers and explicit relationships among files. Table **S26** summarizes the principal distribution layers.

Supplementary Table S26: Release-level organization of MAOMAO resource components.

**Resource layer**	**Directory**	**Main content**
Core Layer	core_layer/	Final sequence-level pivot, source- and endpoint-level processed data, ambiguity and hierarchy audits, quantitative toxicity measurements, quantitative-property cross-reference records, metadata, and sequence-level profiles.
Descriptor Layer	descriptor_layer/	Global sequence-descriptor matrix containing 41 physicochemical and sequence-derived features and accompanying metadata.
Embedding Layer	embedding_layer/	Ten protein language model embedding matrices, one one-hot baseline representation, and their sequence-correspondence files.
Benchmark Layer	benchmark_layer/	Endpoint-specific, representation-independent random and stratified five-fold partitions, identifier–label files, parameters, reports, summaries, completion records, and execution logs.
Documentation Layer	documentation_layer/	Controlled-vocabulary descriptions, metadata schema, licensing and versioning documentation, release manifest, source-license audit, checksums, and results generated by the practical MAOMAO usage examples.

The executable workflows, configuration files, source code, and notebooks used to construct and reuse the resource are maintained in the project GitHub repository. These repository resources complement the archived data layers by providing reproducible procedures for parsing, integration, hierarchy application, sequence characterization, numerical-representation generation, benchmark partitioning, quantitative-property processing, sequence-profile construction, and practical resource reuse.

S5.6 Release, versioning, and licensing

The MAOMAO resource described in this work is distributed through Zenodo as MAOMAO v1.1.0 (DOI: `https://doi.org/10.5281/zenodo.22312156`). The archived resource includes the harmonized peptide-toxicity data, provenance and audit records, structured quantitative toxicity measurements, sequence-level profiles, sequence-derived descriptors, numerical sequence representations, benchmark partitions, metadata, and supporting documentation.

MAOMAO-derived data products are distributed under the Creative Commons Attribution 4.0 International license. Original third-party source materials remain subject to the licensing terms and conditions established by their respective providers. Source code, notebooks, configuration files, and reproducible workflows are distributed through the project GitHub repository under the MIT License.

S6 Demonstration notebooks and worked examples

MAOMAO provides five executable notebooks designed as practical examples for external users of the resource. These notebooks operate directly on the distributed MAOMAO files rather than reconstructing the resource from the original source datasets. Their purpose is to demonstrate resource access, exploratory characterization, identifier-based linkage among resource layers, and minimal downstream computational reuse. Generated figures and tables are written to a dedicated results directory and are kept separate from the Core, Descriptor, Embedding, and Benchmark Layer files.

S6.1 Notebook scope and organization

Table **S27** summarizes the five worked examples. The recommended order is pedagogical rather than computational; the notebooks are self-contained examples and do not require outputs generated by earlier notebooks.

Supplementary Table S27: Demonstration notebooks provided with MAOMAO.

Notebook	Demonstrated operations	Principal resource components
`00_getting_started.ipynb`	Locate and validate distributed assets; load the master sequence resource; inspect evidence states and metadata; query peptides; discover numerical representations and benchmark partitions; and link resources through the stable `id`.	Core; optional Embedding and Benchmark Layers
`01_resource_characterization.ipynb`	Descriptive characterization of sequence lengths, endpoint-state composition, binary class balance, positive endpoint co-annotation, hierarchy consistency, representation coverage, and benchmark inventory.	Core; optional Embedding and Benchmark Layers
`02_neurotoxicity_classification.ipynb`	Reuse one neurotoxicity train–validation–test partition and one protein language model representation to fit Logistic Regression, Linear SVM, and Random Forest models using fixed settings.	Core, Embedding, and Benchmark Layers
`03_quantitative_activity_regression.ipynb`	Inventory numerical toxicity properties, select one homogeneous endpoint–unit subset, map sequences to MAOMAO identifiers, reuse a protein language model representation, and compare a median baseline with Ridge regression.	Core and Embedding Layers
`04_sequence_card_exploration.ipynb`	Stream, retrieve, inspect, and filter sequence profiles from the compressed JSON Lines collection.	Core sequence profiles

S6.2 Illustrative resource characterization and neurotoxicity classification

Figure **S1** combines representative outputs from the descriptive characterization and neurotoxicity-classification notebooks. Panel A shows the sequence-length distribution of the 71,857 Core peptide entities; lengths range from 5 to 70 residues and the median is 25 amino acids. Panels B and C use one stratified neurotoxicity partition as an illustrative supervised-learning example. The notebook links partition membership to a protein language model representation through the stable sequence identifier and evaluates three conventional classifiers without hyperparameter optimization.

On the illustrated test partition, Logistic Regression obtained a balanced accuracy of 0.91, F1 score of 0.90, MCC of 0.81, and ROC AUC of 0.95; Linear SVM obtained 0.87, 0.87, 0.75, and 0.94, respectively; and

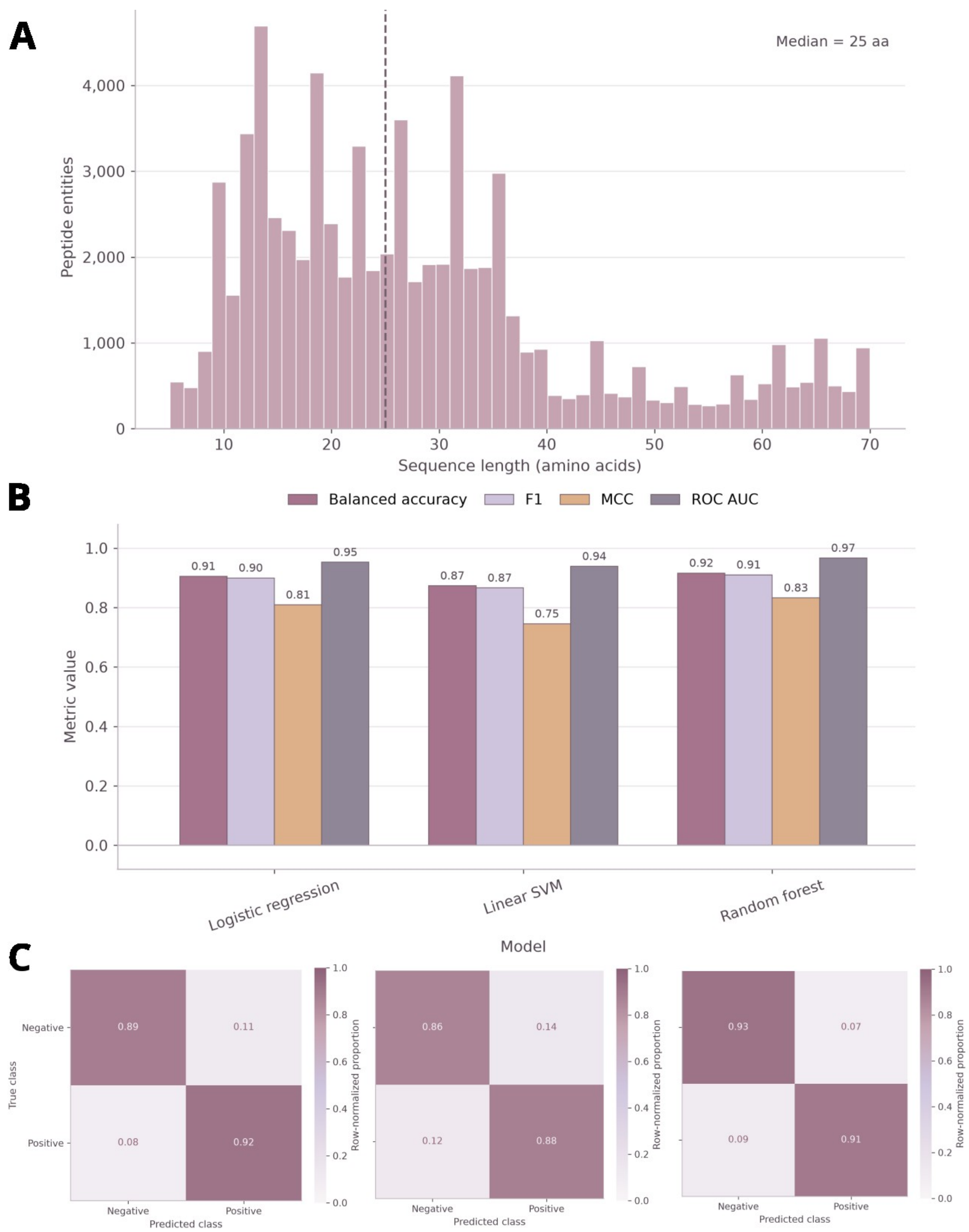

Supplementary Figure S1: Representative outputs from the MAOMAO practical-use notebooks. (A) Sequence-length distribution for the 71,857 Core peptide entities generated by `01_resource_characterization.ipynb`; the dashed line marks the median length of 25 amino acids. (B) Test-set balanced accuracy, F1 score, Matthews correlation coefficient (MCC), and ROC AUC for Logistic Regression, Linear SVM, and Random Forest in the illustrative neurotoxicity-classification example. (C) Row-normalized test-set confusion matrices for the same models, shown from left to right as Logistic Regression, Linear SVM, and Random Forest. Rows denote true classes and columns denote predicted classes. These results illustrate direct reuse of a predefined benchmark partition and numerical representation and are not intended as an optimized MAOMAO benchmark.

Random Forest obtained 0.92, 0.91, 0.83, and 0.97. The row-normalized confusion matrices show negative- and positive-class recalls of 0.89 and 0.92 for Logistic Regression, 0.86 and 0.88 for Linear SVM, and 0.93 and 0.91 for Random Forest. These values correspond to a single worked example and should not be interpreted as performance estimates across the complete 30-seed Benchmark Layer.

S6.3 Illustrative quantitative activity regression

The quantitative regression notebook demonstrates how numerical toxicity properties can be reused without pooling measurements from different endpoints or incompatible units. The notebook first inventories HC50, LC50, LD50, and MHC records and their associated units and then selects one endpoint–unit combination with sufficient observations for an illustrative regression workflow.

The selected example uses LC50 measurements reported in μM, comprising 30 unique peptide sequences. All 30 sequences were mapped to stable MAOMAO identifiers. Repeated measurements were aggregated by exact sequence using the median, and the regression target was transformed as $\log_{10}$(activity value). The numerical predictors were obtained from the ESM2 t6 8M UR50D representation, which provides 320 features per sequence. A deterministic 80:20 train–test split using random state 42 produced 24 training sequences and 6 test sequences.

Table **S28** reports the resulting test metrics. On this small held-out subset, the median baseline outperformed the fixed Ridge model, and the Ridge model produced a strongly negative R^2. These results are retained because the notebook is intended to demonstrate a transparent quantitative-data reuse workflow rather than to present an optimized regression model.

Supplementary Table S28: Test-set results for the illustrative LC50 regression workflow. MAE and RMSE are reported on the $\log_{10}$-transformed activity scale; $n_{\text{test}} = 6$. Spearman correlation is undefined for the median baseline because it produces a constant prediction.

Model	MAE	RMSE	R^2	Spearman ρ	Spearman p
Median baseline	0.327	0.412	-0.030	—	—
Ridge ($\alpha = 1$)	1.146	1.393	-10.754	0.314	0.544

S6.4 Sequence-profile exploration and output separation

The final notebook provides a deliberately lightweight example for exploring the sequence-profile collection. It demonstrates compressed JSON Lines access, retrieval of an individual profile through its stable MAOMAO identifier, inspection of final endpoint states and supporting evidence, and simple endpoint- and status-based filtering. The profiles complement the compact sequence-level pivot by collecting endpoint evidence summaries, ontology relationships, toxicity targets, numerical toxicity properties, source provenance, and selected physicochemical information within a single inspectable record.

The worked examples use the distributed MAOMAO files as read-only analytical inputs. Generated tables and figures are stored separately under `documentation_layer/results_how_to_use_maomao/` and do not modify the Core, Descriptor, Embedding, or Benchmark Layer resources. This separation allows the notebooks to demonstrate direct reuse of MAOMAO while preserving the distributed resource files unchanged.

References

Ibrahim Abdelbaky, Mohamed Elhakeem, Hilal Tayara, Elsayed Badr, and Mustafa Abdul Salam. Enhanced prediction of hemolytic activity in antimicrobial peptides using deep learning-based sequence analysis. *BMC bioinformatics*, 25(1):368, 2024.

Sultan Almotairi, Elsayed Badr, Ibrahim Abdelbaky, Mohamed Elhakeem, and Mustafa Abdul Salam. Hybrid transformer-cnn model for accurate prediction of peptide hemolytic potential. *Scientific Reports*, 14 (1):14263, 2024.

Hazem Alsamkary, Mohamed Elshaffei, Mohamed Elkerdawy, and Ahmed Elnaggar. Ankh3: Multi-task pretraining with sequence denoising and completion enhances protein representations. *arXiv preprint arXiv:2505.20052*, 2025.

Mehrad Ansari and Andrew D White. Learning peptide properties with positive examples only. *bioRxiv*, pages 2023–06, 2023a.

Mehrad Ansari and Andrew D White. Serverless prediction of peptide properties with recurrent neural networks. *Journal of Chemical Information and Modeling*, 63(8):2546–2553, 2023b.

Apache Software Foundation. Apache license, version 2.0, 2004. URL `https://www.apache.org/licenses/LICENSE-2.0`. Consultado: 27 de julio de 2026.

Özlem Sultan Aslantürk. In vitro cytotoxicity and cell viability assays: principles, advantages, and disadvantages. In *Genotoxicity-A predictable risk to our actual world*. IntechOpen, 2017.

Nancy Baker, Alan Boobis, Lyle Burgoon, Edward Carney, Richard Currie, Ellen Fritsche, Thomas Knudsen, Madeleine Laffont, Aldert H Piersma, Alan Poole, et al. Building a developmental toxicity ontology. *Birth defects research*, 110(6):502–518, 2018.

Kathryn Bambino and Jaime Chu. Zebrafish in toxicology and environmental health. *Current topics in developmental biology*, 124:331–367, 2017.

Pranshul Bhatnagar, Yashi Khandelwal, Shagun Mishra, Arnab Dutta, Debirupa Mitra, Swati Biswas, et al. Predicting antibacterial activity, efficacy, and hemotoxicity of peptides using an explainable machine learning framework. *Process Biochemistry*, 145:163–174, 2024.

Edoardo Bizzotto, Guido Zampieri, Laura Treu, Pasquale Filannino, Raffaella Di Cagno, and Stefano Campanaro. Classification of bioactive peptides: a systematic benchmark of models and encodings. *Computational and Structural Biotechnology Journal*, 23:2442–2452, 2024.

Gabriel Cabas-Mora, Anamaría Daza, Nicole Soto-García, Valentina Garrido, Diego Alvarez, Marcelo Navarrete, Lindybeth Sarmiento-Varón, Julieta H Sepúlveda Yañez, Mehdi D Davari, Frederic Cadet, et al. Peptipedia v2. 0: a peptide sequence database and user-friendly web platform. a major update. *Database*, 2024:baae113, 2024.

Salvatore Candido, Thomas Hayes, Alexander Derry, Roshan Rao, Zeming Lin, Robert Verkuil, Bryan Wu, Jin Sub Lee, Elise S. Bruguera, Jehan A. Keval, Mykhailo Kopylov, John E. Pak, Wesley Wu, Neil Thomas, Samson Mataraso, Alvin Hsu, Ashton C. Trotman-Grant, Kilian Fatras, Allan dos Santos Costa, Rohil Badkundri, Halil Akın, Deniz Oktay, Jonathan Deaton, Elizabeth Montabana, Hrishita Sitwala, Yue Yu, Marius Wiggert, Dylan Alexander Carlin, Anthony W. Goering, Tomasz Blazejewski, McCullen Sandora, Michael Hla, Tina Z. Jia, Leon H. Kloker, Nicholas J. Sofroniew, Masatoshi Uehara, Jassi Pannu, Sharrol Bachas, Daniel S. Liu, Tom Sercu, and Alexander Rives. Language modeling materializes a world model of protein biology, 2026. URL `https://www.biorxiv.org/content/10.64898/2026.06.03.729735`. Preprint.

Kevin Castillo-Mendieta, Guillermin Aguero-Chapin, Edgar Marquez, Yunierkis Perez-Castillo, Stephen J Barigye, Mariela Pérez-Cárdenas, Facundo Peréz-Giménez, and Yovani Marrero-Ponce. Multiquery similarity searching models: an alternative approach for predicting hemolytic activity from peptide sequence. *Chemical Research in Toxicology*, 37(4):580–589, 2024a.

Kevin Castillo-Mendieta, Guillermin Agüero-Chapin, Edgar A Marquez, Yunierkis Perez-Castillo, Stephen J Barigye, Nelson Santiago Vispo, Cesar R García-Jacas, and Yovani Marrero-Ponce. Peptide hemolytic activity analysis using visual data mining of similarity-based complex networks. *npj Systems Biology and Applications*, 10(1):115, 2024b.

Kumardeep Chaudhary, Ritesh Kumar, Sandeep Singh, Abhishek Tuknait, Ankur Gautam, Deepika Mathur, Priya Anand, Grish C Varshney, and Gajendra PS Raghava. A web server and mobile app for computing hemolytic potency of peptides. *Scientific reports*, 6(1):22843, 2016.

Sih-Han Chen, Jen-Chieh Yu, Yi-Hsiang Lin, Shao-Chun Kuo, Kuan Ni, and Ching-Tai Chen. Hepad: enhancing hemolytic peptide prediction with adaptive feature engineering and diverse sequence descriptors. *BMC bioinformatics*, 26(1):234, 2025.

Creative Commons. Creative commons attribution 4.0 international public license, 2013. URL `https://creativecommons.org/licenses/by/4.0/legalcode`. Consultado: 27 de julio de 2026.

Durdam Das, Mohini Jaiswal, Fatima Nazish Khan, Shahzaib Ahamad, and Shailesh Kumar. Plantpepdb: A manually curated plant peptide database. *Scientific reports*, 10(1):2194, 2020.

Zhenjiao Du, Xingjian Ding, Yixiang Xu, and Yonghui Li. Unidl4biopep: a universal deep learning architecture for binary classification in peptide bioactivity. *Briefings in Bioinformatics*, 24(3):bbad135, 2023.

Hossein Ebrahimikondori, Darcy Sutherland, Anat Yanai, Amelia Richter, Ali Salehi, Chenkai Li, Lauren Coombe, Monica Kotkoff, René L Warren, and Inanc Birol. Structure-aware deep learning model for peptide toxicity prediction. *Protein Science*, 33(7):e5076, 2024.

Ahmed Elnaggar, Michael Heinzinger, Christian Dallago, Ghalia Rehawi, Yu Wang, Llion Jones, Tom Gibbs, Tamas Feher, Christoph Angerer, Martin Steinegger, et al. Prottrans: toward understanding the language of life through self-supervised learning. *IEEE transactions on pattern analysis and machine intelligence*, 44(10):7112–7127, 2021.

Ahmed Elnaggar, Hazem Essam, Wafaa Salah-Eldin, Walid Moustafa, Mohamed Elkerdawy, Charlotte Rochereau, and Burkhard Rost. Ankh: Optimized protein language model unlocks general-purpose modelling. *arXiv preprint arXiv:2301.06568*, 2023.

Free Software Foundation. GNU general public license, version 3, 2007. URL `https://www.gnu.org/licenses/gpl-3.0.html`. Consultado: 27 de julio de 2026.

Shun Gao, Yanna Jia, Feifei Cui, Junlin Xu, Yajie Meng, Leyi Wei, Qingchen Zhang, Quan Zou, and Zilong Zhang. Plptp: a motif-based interpretable deep learning framework based on protein language models for peptide toxicity prediction. *Journal of Molecular Biology*, 437(12):169115, 2025.

Ankur Gautam, Kumardeep Chaudhary, Sandeep Singh, Anshika Joshi, Priya Anand, Abhishek Tuknait, Deepika Mathur, Grish C Varshney, and Gajendra PS Raghava. Hemolytik: a database of experimentally determined hemolytic and non-hemolytic peptides. *Nucleic acids research*, 42(D1):D444–D449, 2014.

Alexander GB Grønning, Tim Kacprowski, and Camilla Scheele. Multipep: a hierarchical deep learning approach for multi-label classification of peptide bioactivities. *Biology Methods and Protocols*, 6(1):bpab021, 2021.

Jiahui Guan, Peilin Xie, Dian Meng, Lantian Yao, Dan Yu, Ying-Chih Chiang, Tzong-Yi Lee, and Junwen Wang. Toxipep: Peptide toxicity prediction via fusion of context-aware representation and atomic-level graph. *Computational and Structural Biotechnology Journal*, 27:2347–2358, 2025.

Sudheer Gupta, Pallavi Kapoor, Kumardeep Chaudhary, Ankur Gautam, Rahul Kumar, and Gajendra PS Raghava. In silico approach for predicting toxicity of peptides and proteins. *PloS one*, 8(9):e73957–e73957, 2013.

Jiyun Han, Tongxin Kong, and Juntao Liu. Pepnet: an interpretable neural network for anti-inflammatory and antimicrobial peptides prediction using a pre-trained protein language model. *Communications Biology*, 7(1):1198, 2024.

Md Mehedi Hasan, Nalini Schaduangrat, Shaherin Basith, Gwang Lee, Watshara Shoombuatong, and Balachandran Manavalan. Hlppred-fuse: improved and robust prediction of hemolytic peptide and its activity by fusing multiple feature representation. *Bioinformatics*, 36(11):3350–3356, 2020.

Jesús Herrera-Bravo, Jorge G Farías, Fernanda Parraguez Contreras, Lisandra Herrera-Belén, and Jorge F Beltrán. Pep-predna+: A web server for prediction of highly specific peptides targeting voltage-gated na+ channels using machine learning techniques. *Computers in Biology and Medicine*, 145:105414, 2022.

Cataia Ives, Ivana Campia, Rong-Lin Wang, Clemens Wittwehr, and Stephen Edwards. Creating a structured adverse outcome pathway knowledgebase via ontology-based annotations. *Applied in vitro toxicology*, 3 (4):298–311, 2017.

Aashish Jain and Daisuke Kihara. Nntox: gene ontology-based protein toxicity prediction using neural network. *Scientific reports*, 9(1):17923, 2019.

Shihu Jiao, Xiucai Ye, Tetsuya Sakurai, Quan Zou, and Ruijun Liu. Integrated convolution and self-attention for improving peptide toxicity prediction. *Bioinformatics*, 40(5):btae297, 2024.

Shihu Jiao, Xiucai Ye, Tetsuya Sakurai, Quan Zou, Wu Han, and Chao Zhan. Integration of pre-trained protein language models with equivariant graph neural networks for peptide toxicity prediction. *BMC biology*, 23(1):229, 2025.

Xiaoyang Jing, Qiwen Dong, Daocheng Hong, and Ruqian Lu. Amino acid encoding methods for protein sequences: a comprehensive review and assessment. *IEEE/ACM transactions on computational biology and bioinformatics*, 17(6):1918–1931, 2019.

Dmitry A Karasev, Georgii S Malakhov, and Boris N Sobolev. Quantitative prediction of hemolytic activity of peptides. *Computational Toxicology*, 32:100335, 2024.

Hossein Khabbaz, Mohammad Hossein Karimi-Jafari, Ali Akbar Saboury, and Bagher BabaAli. Prediction of antimicrobial peptides toxicity based on their physico-chemical properties using machine learning techniques. *BMC bioinformatics*, 22(1):549, 2021.

KrenAI-Lab. BioSieve: Redundancy reduction and leakage-aware dataset partitioning for biological machine learning. `https://pypi.org/project/biosieve/`, 2026a. Python package, version 0.1.0, accessed 12 May 2026.

KrenAI-Lab. roxy. https://github.com/kren-ai-lab/roxy, 2026b. GitHub repository.

KrenAI-Lab. Sylphy: Protein sequence representation: encoders, embeddings, and reductions. https://pypi.org/project/sylphy/, 2026c. Python package, version 0.2.0, accessed 12 May 2026.

Ashok Kumar, Sonia Chadha, Mradul Sharma, and Mukesh Kumar. Deciphering optimal molecular determinants of non-hemolytic, cell-penetrating antimicrobial peptides through bioinformatics and random forest. *Briefings in Bioinformatics*, 26(1):bbaf049, 2025.

Yanru Li, Liming Gao, Wenqian Zhao, Ziyu Wang, Xianghan Xu, Jinwen Ji, Yuan Zhang, Yufeng Liu, Liangyun Zhang, Yong Yang, et al. Aipampds: an ai platform for antimicrobial peptide design and screening. *bioRxiv*, pages 2025–03, 2025.

Zeming Lin, Halil Akin, Roshan Rao, Brian Hie, Zhongkai Zhu, Wenting Lu, Nikita Smetanin, Allan dos Santos Costa, Maryam Fazel-Zarandi, Tom Sercu, Sal Candido, et al. Language models of protein sequences at the scale of evolution enable accurate structure prediction. *bioRxiv*, 2022.

Hongwu Lv, Ke Yan, and Bin Liu. Tppred-le: therapeutic peptide function prediction based on label embedding. *BMC biology*, 21(1):238, 2023.

Tianyue Ma, Yanchao Liu, Bingxin Yu, Xin Sun, Huiyuan Yao, Chen Hao, Jianhui Li, Maryam Nawaz, Xun Jiang, Xingzhen Lao, et al. Dramp 4.0: an open-access data repository dedicated to the clinical translation of antimicrobial peptides. *Nucleic Acids Research*, 53(D1):D403–D410, 2025.

Katsumi Matsuzaki. Control of cell selectivity of antimicrobial peptides. *Biochimica et Biophysica Acta (BBA)-Biomembranes*, 1788(8):1687–1692, 2009.

Charlene McQueen. *Comprehensive toxicology*. Elsevier, 2017.

Piotr Minkiewicz, Anna Iwaniak, and Małgorzata Darewicz. Biopep-uwm database of bioactive peptides: Current opportunities. *International journal of molecular sciences*, 20(23):5978, 2019.

Rajat Kumar Mondal, Debarup Sen, Ankish Arya, and Sintu Kumar Samanta. Developing anti-microbial peptide database version 1 to provide comprehensive and exhaustive resource of manually curated amps. *Scientific Reports*, 13(1):17843, 2023.

Vladimir Morozov, Carlos HM Rodrigues, and David B Ascher. Csm-toxin: a web-server for predicting protein toxicity. *Pharmaceutics*, 15(2):431, 2023.

Holly M Mortensen, Marvin Martens, Jonathan Senn, Trevor Levey, Chris T Evelo, Egon L Willighagen, and Thomas Exner. The aop-db rdf: applying fair principles to the semantic integration of aop data using the research description framework. *Frontiers in Toxicology*, 4:803983, 2022.

Raphaël Mourad. Mistral-prot-v1-134m (mistral for protein). https://huggingface.co/RaphaelMourad/Mistral-Prot-v1-134M, 2024. Hugging Face Model Repository.

Open Data Commons. Open data commons database contents license (DbCL) v1.0, 2008. URL https://opendatacommons.org/licenses/dbcl/1-0/. Consultado: 27 de julio de 2026.

Open Data Commons. Open data commons open database license (ODbL) v1.0, 2009. URL https://opendatacommons.org/licenses/odbl/1-0/. Consultado: 27 de julio de 2026.

Open Source Initiative. The MIT license, 2026. URL https://opensource.org/licenses/MIT. Consultado: 27 de julio de 2026.

AnQi Ouyang, Fang Ge, Jiangning Song, and Dong-Jun Yu. De novo design of functionally diverse and druggable antimicrobial peptides by diffusion and multimodal deep learning. *bioRxiv*, pages 2025–07, 2025.

Xiaoyong Pan, Jasper Zuallaert, Xi Wang, Hong-Bin Shen, Elda Posada Campos, Denys O Marushchak, and Wesley De Neve. Toxdl: deep learning using primary structure and domain embeddings for assessing protein toxicity. *Bioinformatics*, 36(21):5159–5168, 2020.

Gulnaz Perveen, Fahad Alturise, Tamim Alkhalifah, and Yaser Daanial Khan. Hemolytic-pred: a machine learning-based predictor for hemolytic proteins using position and composition-based features. *Digital Health*, 9:20552076231180739, 2023.

Fabien Plisson, Obed Ramírez-Sánchez, and Cristina Martínez-Hernández. Machine learning-guided discovery and design of non-hemolytic peptides. *Scientific reports*, 10(1):16581, 2020.

Peng Qiu, Hanqi Feng, Meng-Chun Zhang, and Barnabas Poczos. Amplyze: A deep learning model for predicting the hemolytic concentration. *arXiv preprint arXiv:2507.08162*, 2025.

Anand Singh Rathore, Akanksha Arora, Shubham Choudhury, Purva Tijare, and Gajendra PS Raghava. Toxinpred 3.0: An improved method for predicting the toxicity of peptides. *BioRxiv*, pages 2023–08, 2023.

Anand Singh Rathore, Saloni Jain, Shubham Choudhury, and Gajendra PS Raghava. Ntxpred2: a large language model for predicting neurotoxic peptides and neurotoxins. *bioRxiv*, 2025a.

Anand Singh Rathore, Nishant Kumar, Shubham Choudhury, Naman Kumar Mehta, and Gajendra PS Raghava. Prediction of hemolytic peptides and their hemolytic concentration. *Communications Biology*, 8(1):176, 2025b.

Sudipto Saha and Gajendra PS Raghava. Prediction of neurotoxins based on their function and source. *In silico biology*, 7(4-5):369–387, 2007.

Milad Salem, Arash Keshavarzi Arshadi, and Jiann Shiun Yuan. Ampdeep: hemolytic activity prediction of antimicrobial peptides using transfer learning. *BMC bioinformatics*, 23:1, 2022.

Harshika Sharma, Mayank Singh Thakur, Avinash Barala, Mohd Saad Khan, Sumit Bhagat, and Ganesh Bagler. Multitox: A sequence-based stacked ensemble model for multiclass protein toxin classification. *International Journal of Biological Macromolecules*, 327:147399, 2025.

Neelam Sharma, Leimarembi Devi Naorem, Shipra Jain, and Gajendra PS Raghava. Toxinpred2: an improved method for predicting toxicity of proteins. *Briefings in bioinformatics*, 23(5):bbac174, 2022.

Hua Shi, Yan Li, Yi Chen, Yuming Qin, Yifan Tang, Xun Zhou, Ying Zhang, and Yun Wu. Toxmva: An end-to-end multi-view deep autoencoder method for protein toxicity prediction. *Computers in Biology and Medicine*, 151:106322, 2022.

Ayushi Singh, Kavin Raj SA, Anand Singh Rathore, and Gajendra P. S. Raghava. Hemolytik2: An updated database of hemolytic peptides and proteins. *bioRxiv*, 2025. doi: 10.1101/2025.05.12.653624. URL `https://www.biorxiv.org/content/early/2025/05/14/2025.05.12.653624`.

Ayushi Singh, Kavin Raj Sa, Anand Singh Rathore, and Gajendra PS Raghava. Hemolytik 2: An updated database of hemolytic peptides and proteins. *Chemical Research in Toxicology*, 39(2):208–215, 2026.

Sandeep Singh, Kumardeep Chaudhary, Sandeep Kumar Dhanda, Sherry Bhalla, Salman Sadullah Usmani, Ankur Gautam, Abhishek Tuknait, Piyush Agrawal, Deepika Mathur, and Gajendra PS Raghava. Satpdb: a database of structurally annotated therapeutic peptides. *Nucleic acids research*, 44(D1):D1119–D1126, 2016.

B Logan Spaller, Julie M Trieu, and Paulo F Almeida. Hemolytic activity of membrane-active peptides correlates with the thermodynamics of binding to 1-palmitoyl-2-oleoyl-sn-glycero-3-phosphocholine bilayers. *The Journal of membrane biology*, 246(3):257–262, 2013.

Sebastian Spänig, Siba Mohsen, Georges Hattab, Anne-Christin Hauschild, and Dominik Heider. A large-scale comparative study on peptide encodings for biomedical classification. *NAR genomics and bioinformatics*, 3(2):lqab039, 2021.

Zhelyazko Terziyski, Margarita Terziyska, Ivelina Deseva, Stanka Hadzhikoleva, Albert Krastanov, Dasha Mihaylova, and Emil Hadzhikolev. Peplab platform: Database and software tools for analysis of food-derived bioactive peptides. *Applied Sciences*, 13(2):961, 2023.

Patrick Brendan Timmons and Chandralal M Hewage. Happenn is a novel tool for hemolytic activity prediction for therapeutic peptides which employs neural networks. *Scientific reports*, 10(1):10869, 2020.

Andrey A Toropov, Alla P Toropova, Valentin O Kudyshkin, Emilio Benfenati, Danuta Leszczynska, and Jerzy Leszczynski. Simulation peptide toxicity using the fragments of local symmetry in amino acid sequences. *BioSystems*, page 105554, 2025.

Duong Thanh Tran, Nhat Truong Pham, Nguyen Doan Hieu Nguyen, Leyi Wei, and Balachandran Manavalan. Hypeptox-fuse: An interpretable hybrid framework for accurate peptide toxicity prediction fusing protein language model-based embeddings with conventional descriptors. *Journal of Pharmaceutical Analysis*, page 101410, 2025.

Hao Wan, Qing Liu, and Ying Ju. Utilize a few features to classify presynaptic and postsynaptic neurotoxins. *Computers in Biology and Medicine*, 152:106380, 2023.

Feng Wang, Peilin Xie, Xingqiao Lin, Jiahui Guan, Chang Liu, Xuxin He, Tzong-Yi Lee, Leyi Wei, Xiangrong Liu, and Lantian Yao. Bitoxnet: a deep learning framework integrating multimodal features for accurate identification of neurotoxic peptides and proteins. *BMC biology*, 24(1):89, 2026.

Jen-Hung Wang and Ting-Yi Sung. Toxteller: predicting peptide toxicity using four different machine learning approaches. *ACS omega*, 9(29):32116–32123, 2024.

Li Wang, Xiangzheng Fu, Jiahao Yang, Xinyi Zhang, Xiucai Ye, Yiping Liu, Tetsuya Sakurai, and Xiangxiang Zeng. Moformer: Multi-objective antimicrobial peptide generation based on conditional transformer joint multi-modal fusion descriptor. *arXiv preprint arXiv:2406.02610*, 2024a.

Li Wang, Yiping Liu, Xiangzheng Fu, Xiucai Ye, Junfeng Shi, Gary G Yen, Quan Zou, Xiangxiang Zeng, and Dongsheng Cao. Hmamp: designing highly potent antimicrobial peptides using a hypervolume-driven multiobjective deep generative model. *Journal of Medicinal Chemistry*, 68(8):8346–8360, 2025a.

Yanling Wang, Na Li, Xiao Wang, Feng Cao, Shuwen Xiong, and Leyi Wei. Integrating protein language models and geometric deep learning for peptide toxicity prediction. *Journal of Chemical Information and Modeling*, 65(14):7800–7810, 2025b.

Yue Wang, Haifan Gong, Xiaojuan Li, Lixiang Li, Yinuo Zhao, Peijing Bao, Qingzhou Kong, Boyao Wan, Yumeng Zhang, Jinghui Zhang, et al. De novo multi-mechanism antimicrobial peptide design via multimodal deep learning. *BioRxiv*, pages 2024–01, 2024b.

Lesong Wei, Xiucai Ye, Yuyang Xue, Tetsuya Sakurai, and Leyi Wei. Atse: a peptide toxicity predictor by exploiting structural and evolutionary information based on graph neural network and attention mechanism. *Briefings in bioinformatics*, 22(5):bbab041, 2021.

Lesong Wei, Xiucai Ye, Tetsuya Sakurai, Zengchao Mu, and Leyi Wei. Toxibtl: prediction of peptide toxicity based on information bottleneck and transfer learning. *Bioinformatics*, 38(6):1514–1524, 2022.

William C Wimley. Describing the mechanism of antimicrobial peptide action with the interfacial activity model. *ACS chemical biology*, 5(10):905–917, 2010.

Thet Su Win, Aijaz Ahmad Malik, Virapong Prachayasittikul, Jarl E S Wikberg, Chanin Nantasenamat, and Watshara Shoombuatong. Hemopred: a web server for predicting the hemolytic activity of peptides. *Future medicinal chemistry*, 9(3):275–291, 2017.

Peilin Xie, Lantian Yao, Jiahui Guan, Chia-Ru Chung, Zhihao Zhao, Feiyu Long, Zhenglong Sun, Tzong-Yi Lee, and Ying-Chih Chiang. Consamphemo: A computational framework for predicting hemolysis of antimicrobial peptides based on machine learning approaches. *Protein Science*, 34(7):e70087, 2025.

Jing Xu, Fuyi Li, Chen Li, Xudong Guo, Cornelia Landersdorfer, Hsin-Hui Shen, Anton Y Peleg, Jian Li, Seiya Imoto, Jianhua Yao, et al. iampcn: a deep-learning approach for identifying antimicrobial peptides and their functional activities. *Briefings in Bioinformatics*, 24(4):bbad240, 2023.

Sen Yang and Piao Xu. Hemodl: Hemolytic peptides prediction by double ensemble engines from rich sequence-derived and transformer-enhanced information. *Analytical Biochemistry*, 690:115523, 2024.

Yang Yang, Haohan Zhang, and Mauno Vihinen. Protoxin, a predictor of protein toxicity. *Toxins*, 17(10): 489, 2025.

Adiba Yaseen, Sadaf Gull, Naeem Akhtar, Imran Amin, and Fayyaz Minhas. Hemonet: Predicting hemolytic activity of peptides with integrated feature learning. *Journal of Bioinformatics and Computational Biology*, 19(05):2150021, 2021. doi: 10.1142/S0219720021500219. URL https://doi.org/10.1142/S0219720021500219. PMID: 34353244.

Qiule Yu, Zhixing Zhang, Guixia Liu, Weihua Li, and Yun Tang. Toxgin: an in silico prediction model for peptide toxicity via graph isomorphism networks integrating peptide sequence and structure information. *Briefings in Bioinformatics*, 25(6):bbae583, 2024.

Shengli Zhang, Jingyi Ren, and Yunyun Liang. An innovative peptide toxicity prediction model based on multi-scale convolutional neural network and residual connection. *Bioinformatics*, 41(10):btaf462, 2025.

Ya Zhao, Shengli Zhang, and Yunyun Liang. Hemofuse: multi-feature fusion based on multi-head cross-attention for identification of hemolytic peptides. *Scientific Reports*, 14(1):22518, 2024.

Yuhong Zhao, Shijing Wang, Wenyi Fei, Yuqi Feng, Le Shen, Xinyu Yang, Min Wang, and Min Wu. Prediction of anticancer peptides with high efficacy and low toxicity by hybrid model based on 3d structure of peptides. *International journal of molecular sciences*, 22(11):5630, 2021.

Lin Zhu, Yi Fang, Shuting Liu, Hong-Bin Shen, Wesley De Neve, and Xiaoyong Pan. Toxdl 2.0: Protein toxicity prediction using a pretrained language model and graph neural networks. *Computational and Structural Biotechnology Journal*, 27:1538–1549, 2025.

Wen Zhu, Yuxin Guo, and Quan Zou. Prediction of presynaptic and postsynaptic neurotoxins based on feature extraction. *Math Biosci Eng*, 18(5):5943–58, 2021.

Shengxin Zhuang, John Tanner, Yusen Wu, Du Huynh, Wei Liu, Xavier Cadet, Nicolas Fontaine, Philippe Charton, Cedric Damour, Frederic Cadet, et al. Non-hemolytic peptide classification using a quantum support vector machine: S. zhuang et al. *Quantum Information Processing*, 23(11):379, 2024.
